# Predictive value of different glycemic variability indicators for prognosis in critically ill patients: a meta-analysis

**DOI:** 10.3389/fendo.2026.1857523

**Published:** 2026-06-22

**Authors:** Lingling Wu, Jie Zhang, Weihong Shen, Fanglei Xu

**Affiliations:** 1 Graduate Student, School of Medicine, Tongji University, Shanghai, China; 2 Nursing Department, Jinshan Hospital of Fudan University, Shanghai, China; 3 Nursing Department, Tongji Hospital, Shanghai, China

**Keywords:** critical illness, critically ill patients, glycemic variability, mortality, prognosis

## Abstract

**Introduction:**

Glycemic variability (GV) strongly influences prognosis in critically ill patients; however, the optimal GV metric is unclear. This meta-analysis compares the association of various GV measures with clinical outcomes.

**Methods:**

PubMed, Embase, Web of Science, and the Cochrane Library were searched until June 2025 for studies linking GV metrics with prognosis in critically ill patients. The extracted GV metrics included coefficient of variation (CV), standard deviation (SD), and mean amplitude of glycemic excursions (MAGE). The primary outcomes were all-cause mortality (ACM) and major adverse cardiovascular event (MACE). Pooled hazard ratios (HRs) were estimated using a random effects model (REM) or a fixed effects model (FEM).

**Results:**

A total of 31 studies on 98,946 patients were included. Elevated CV was significantly associated with increased 30-day, 90-day, and 1-year ACM. MAGE demonstrated the strongest association with 30-day ACM (HR = 1.50, 95%CI = 1.27–1.78). Elevated SD (HR = 2.45) and MAGE (HR = 2.12) were also associated with an increased risk of MACE. Subgroup analysis further revealed that the impact of an increased CV level on 30-day ACM was greater in non-diabetic (NDM) patients (HR = 1.40) than in diabetic (DM) patients (HR = 1.32).

**Discussion:**

Elevated GV, particularly MAGE and CV, independently predicted both short- and long-term ACM and MACE. MAGE showed a strong association with short-term ACM. However, whether it is superior to other GV metrics warrants further investigation since available studies were limited. Clinicians should therefore place greater emphasis on dynamic GV monitoring and individualized glucose management to improve patient outcomes.

**Systematic review registration:**

https://www.crd.york.ac.uk/prospero/display_record.php?RecordID=1071964, identifier CRD420251071964.

## Introduction

Stress-induced hyperglycemia (SIH), common in glucose metabolism among critically ill patients, frequently occurs in acute stress conditions such as trauma, severe infection, and shock (1). Its pathophysiology is essentially an excessive activation of the neuroendocrine system, which causes the massive release of counter-regulatory hormones such as catecholamines, cortisol, and glucagon, thereby inducing severe insulin resistance and hepatic glycogenolysis and gluconeogenesis. Simultaneously, the increase of pro-inflammatory cytokines further inhibits the insulin signaling pathways, leading to marked fluctuations in blood glucose levels (2).

Compared with static measures such as mean blood glucose, glycemic variability (GV) has garnered widespread attention as a key biomarker that reflects dynamic glucose fluctuations and predicts patient prognosis (3–5). High GV is closely related to increased in-hospital mortality (IHM) and possibly elevates the risk of severe cognitive impairment, arrhythmias, and major adverse cardiovascular event (MACE) (6–8).

Elevated GV levels are independently related to greater risk of all-cause mortality (ACM) in critically ill patients. For instance, high coefficient of variation (CV) and mean amplitude of glycemic excursions (MAGE) significantly increase the risks of IHM and cognitive impairment in intensive care unit (ICU) patients (7–9). In patients with acute cardiovascular and cerebrovascular events, maintaining stable GV confers significant organ-protective effects (6). The pathogenic mechanisms are likely related to oxidative stress, endothelial dysfunction, and systemic inflammatory responses triggered by marked glucose fluctuations. Notably, each one-unit increase in log-transformed CV was associated with an approximately 30% higher IHM risk (7).

Although a recent meta-analysis (10) has explored the association between specific GV metrics and cardiovascular outcomes, a comprehensive evaluation of multiple dynamic GV dimensions in relation to both short- and long-term ACM across broader critically ill populations is still lacking. In current clinical practice, various GV indices, including CV, MAGE, SD, and the stress hyperglycemia ratio (SHR), have been applied for risk stratification in different subgroups of critically ill patients (8, 9, 11). Targeted glucose monitoring and intervention strategies may reduce insulin requirements and improve clinical outcomes (12). However, identifying the GV parameter with the greatest prognostic value remains an important unresolved challenge. Therefore, our study aimed to determine which GV indicators most effectively predict outcomes in critically ill patients and to quantitatively compare the strength of the associations between different GV metrics and clinical endpoints through meta-analysis, providing evidence to support precision-based glucose monitoring strategies.

## Methods

Our study followed the Preferred Reporting Items for Systematic Reviews and Meta-analyses (PRISMA) guidelines (11). The protocol was registered in June 2025 with PROSPERO (CRD420251071964).

### Search strategy

PubMed, Embase, the Cochrane Library, and Web of Science were searched until 9 June 2025 using MeSH and free-text terms. The main search terms included: Critical Illness; Sepsis; Acute Coronary Syndrome; Multiple Organ Failure; Respiratory Distress Syndrome; Shock; Acute Kidney Injury; Multiple Trauma; Blood Glucose or glucose blood level; varia* (for variability). Moreover, the references of eligible and gray literature were manually screened. The strategy is detailed in Document 1 of the **Supplementary Material**.

### Eligibility criteria

The inclusion criteria were: 1) population: critically ill adult patients in the ICU; 2) exposure: reported measurements of GV parameters, including SD, CV, and MAGE; 3) study design: observational or prospective studies; and 4) outcomes: reported the association of GV parameters with patient prognosis, including the 30-day, 90-day, and 1-year ACM, ICU mortality, IHM, and MACE.

The exclusion criteria were: 1) animal or cellular experiments, case reports, conference abstracts, editorials, experimental protocols, letters, and reviews, among others; 2) missing or erroneous data; 3) duplicates; 4) unavailable full text; and 5) overlapping patient populations.

### Literature screening and data extraction

The retrieved literature was uploaded to EndNote for deduplication. Lingling Wu and Weihong Shen independently checked the titles and abstracts against the eligibility criteria, followed by full-text screening. Disagreements between the two independent reviewers were resolved through discussion or arbitration by a third senior reviewer. Data were independently extracted by Lingling Wu and Weihong Shen using a predesigned electronic data extraction form. Extracted information included the first author, publication year, study design, country, sample size, sex and age distribution, baseline characteristics, GV parameters, primary and secondary outcomes, patient type, and diabetes status.

### Quality assessment

Two authors (Lingling Wu and Fanglei Xu) independently assessed the quality of the eligible studies using the Newcastle–Ottawa Scale (NOS) (12), which assesses eight items in three domains: selection, comparability, and exposure. The scale was scored 0–9, with ≥6 and ≤5 indicating high and low quality, respectively.

### Statistical analysis

Statistical analyses were conducted using Stata 18.0. The associations between various GV parameters and prognosis among critically ill patients were examined through hazard ratios (HRs) with 95% confidence intervals (CIs). Maximally adjusted HRs were preferentially extracted to minimize the influence of potential confounding factors. Studies reporting only unadjusted odds ratios (ORs) or relative risks (RRs) without available HRs were excluded to maintain methodological consistency. To account for differences in the scaling of continuous GV metrics, analyses were strictly stratified into continuous (CV_con_) and categorical (CV_cat_) variables. Heterogeneity among studies was assessed using the Cochrane *I*^2^ statistic. A fixed effects model (FEM; inverse-variance method) was applied when the heterogeneity was insignificant (*p* > 0.1 and *I*^2^ ≤ 50%), whereas a random effects model (REM; DerSimonian–Laird method) was used in the presence of substantial heterogeneity (*p* ≤ 0.1 and *I*^2^ > 50%). Subgroup analyses according to diabetes status were performed to further explore the magnitude and potential sources of heterogeneity. Additional subgroup analyses based on primary disease categories (e.g., sepsis, cardiovascular diseases, and neurological diseases) were carried out to investigate clinical heterogeneity. Sensitivity analyses, performed by sequentially excluding individual studies, were used to evaluate the robustness and reliability of the findings. For outcomes including more than 10 studies, publication bias was assessed visually using funnel plots and quantitatively using Egger’s test, with *p* < 0.05 indicating significant publication bias.

## Results

### Literature search and screening

**Figure 1:**
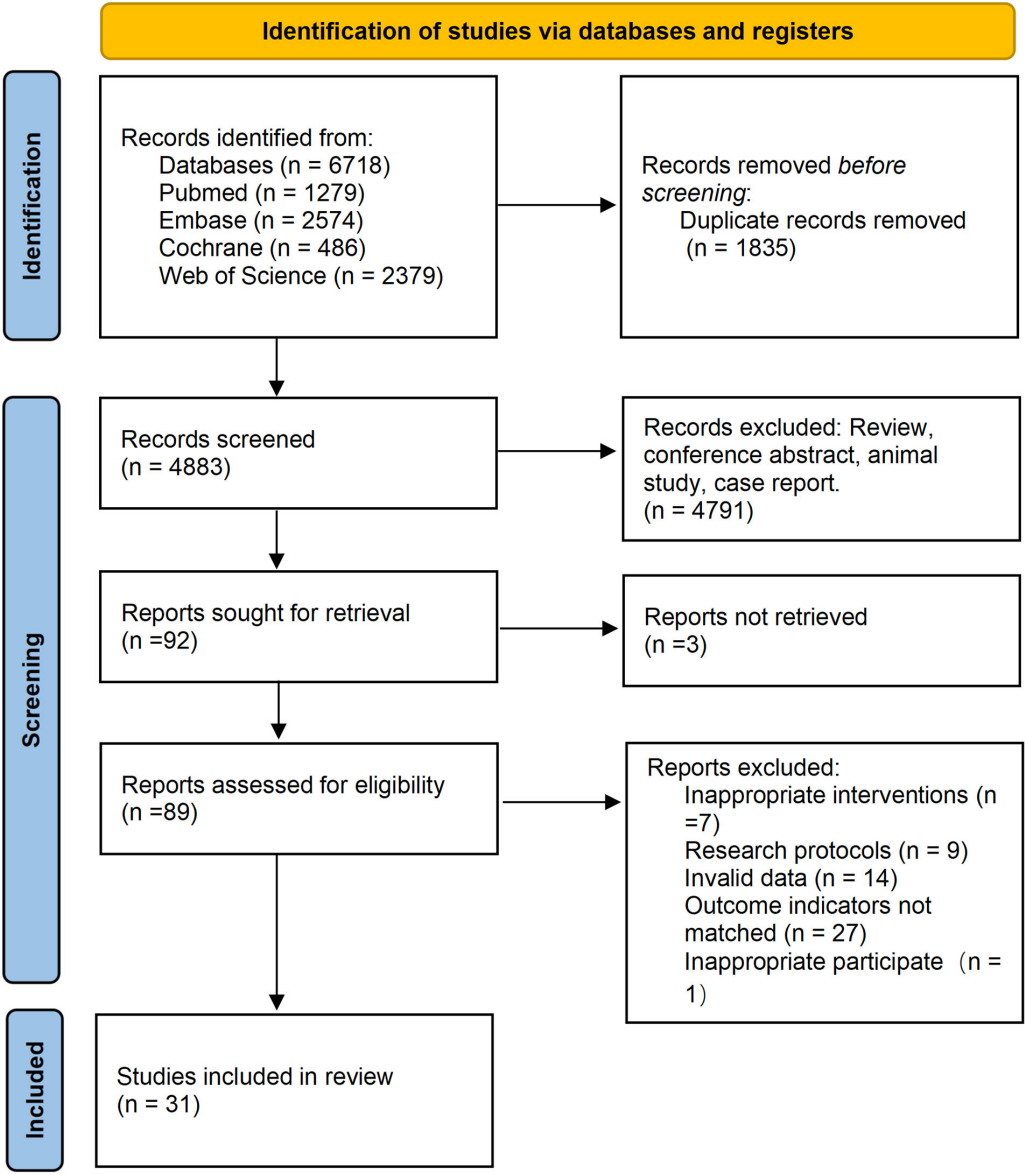
Preferred reporting items for systematic reviews and meta-analyses (PRISMA) flow diagram.

A total of 6,718 records were initially retrieved. After removal of 1,835 duplicates and screening of the titles and abstracts, 4,791 articles were excluded. The remaining studies underwent full-text review according to the eligibility criteria, and 31 studies were ultimately included in the meta-analysis. The study selection process is provided in **Figure 1**.

### Characteristics and quality of the included studies

The 31 included studies (3, 6, 8, 9, 13–39) were conducted across nine countries (China, USA, Australia, France, Brazil, India, Japan, South Korea, and UK) and involved 98,946 patients, including 59,089 men and 39,857 women. The GV parameters evaluated included CV (CV_con_ and CV_cat_, *n* = 22), SD (*n* = 10), and MAGE (*n* = 8). Detailed study characteristics are summarized in **Table 1**. All included studies achieved NOS scores >6, indicating generally high methodological quality. Detailed quality assessment results are presented in Document 2 of the **Supplementary Material**.

**Table 1:** Detailed characteristics of the included cohorts.

Study (year)	Study design	Country	Population	Size	Men	Women	Age, mean (SD)	GV metric	Monitoring method	Outcomes	Diabetes
Boschi et al. (13), 2024	Retrospective	Brazil	COVID-19	239	134	105	58.61 (16.68)	CV	POCT	28-day	YES
Wang et al. (14), 2025	Observational	China	ACD	2,807	1,650	1,157	71.00 (2.85)	CV	NA	28-day, 90-day	YES
Wang et al. (6), 2024	Observational	China	AMI	7,136	5,095	2,041	62.50 (11.90)	SD	Lab FPG	30-day, MACE	YES
Zhu et al. (9), 2025	Retrospective	China	Critically ill patients	13,852	8,808	5,044	66.73 (14.18)	MAGE	POCT	28-day, ICU, IHM	YES
Yu et al. (15), 2025	Retrospective	China	TAVR	3,342	2,162	1,180	70.65 (12.61)	CV	NA	30-day, 1-year	YES
Yang et al. (16), 2025	Retrospective	China	CVD	778	379	399	73.30 (16.34)	CV	NA	90-day, 1-year	NO
Wang et al. (17), 2025	Retrospective	China	CVD	732	396	336	70.65 (15.60)	CV	NA	90-day, 1-year	YES
Shuai et al. (3), 2025	Retrospective	China	HF	8,980	5,024	3,956	73.23 (13.29)	CV	NA	IHM, 1-year	YES
Prakash et al. (18), 2025	Observational	India	Sepsis	80	40	40	45.6 (15.37)	MAGE, SD, CV	POCT	IHM	NO
Hou et al. (19), 2025	Retrospective	China	CVD	1,056	469	587	61.00 (16.33)	CV	NA	30 days, ICU, IHM, 90-day, 1-year	NO
Chen et al. (20), 2024	Retrospective	China	AMI	2,590	1,546	1,044	67.22 (12.40)	CV	NA	IHM	NO
Kim et al. (21), 2022	Retrospective	South Korea	Pneumonia	282	202	80	68.65 (1.70)	CV	Lab FPG	28-day	YES
Chao et al. (22), 2020	Retrospective	China	Sepsis	452	346	106	71.40 (14.70)	MAGE, CV	POCT	30-day	YES
Zhou et al. (23), 2025	Retrospective	China	Sepsis	7,049	4,120	2,929	64.01 (17.24)	CV, SD	POCT	28-day	NO
Liu et al. (24), 2025	Retrospective	China	CVD	2,240	1,142	1,098	65.00 (17.80)	CV	NA	30-day, ICU, IHM, 90-day	NO
Qi et al. (25), 2024	Retrospective	China	TBI	1,641	1,040	601	65.95 (24.48)	CV	NA	IHM	YES
Guo et al. (26), 2024	Retrospective	China	AKI	6,777	3,924	2,853	63.50 (16.70)	CV	NA	30-day	YES
Chen et al. (27), 2024	Retrospective	UK	AF	8,989	5,193	3,796	76.15 (12.31)	CV	NA	30-day, 90-day, 1-year	NO
Cai et al. (8), 2023	Retrospective	China	CVD	4,809	2,567	2,242	70.62 (16.30)	CV	NA	30-day	YES
Lu et al. (28), 2022	Retrospective	China	Sepsis	7,104	3,891	3,213	68.36 (17.60)	CV	NA	ICU	YES
Gerbaud et al. (29), 2022	Observational	France	HF	392	271	121	73.00 (10.20)	SD	POCT	MACE	NO
Su et al. (30), 2021	Prospective	China	ACS	759	465	294	62.80 (9.50)	MAGE	CGMS/SMBG	MACE	NO
Lu et al. (31), 2021	Retrospective	China	SAP	769	423	346	59.51 (19.25)	CV, SD	NA	IHM	NO
Cai et al. (32), 2020	Retrospective	China	CVD	158	100	58	64.86 (9.66)	MAGE, SD, CV	POCT	90-day	NO
Gerbaud et al. (33), 2019	Retrospective	France	ACS	327	252	75	69.00 (11.90)	SD	POCT	MACE	NO
Doola et al. (34), 2019	Prospective	Australia	Critically ill patients	759	499	260	56.13 (18.42)	CV	NA	ICU	NO
Takahashi et al. (35), 2018	Prospective	Japan	ACS	417	348	69	65.30 (13.39)	MAGE	CGMS	MACE	NO
Lanspa et al. (36), 2014	Prospective	USA	Critically ill patients	6,101	3,630	2,471	64.99 (2.96)	CV	POCT	30-day	YES
Ali et al. (37), 2008	Retrospective	USA	Sepsis	1,246	657	589	60.5	MAGE, SD	Lab FPG	IHM	NO
Egi et al. (38), 2006	Retrospective	Australia	Critically ill patients	7,049	4,287	2,762	61.00 (18.00)	SD	POCT	ICU, IHM	NO
Wang et al. (39), 2014	Prospective	China	AMI	34	29	5	62.81 (11.41)	MAGE, SD	CGMS/SMBG	MACE	NO

ACD, atherosclerotic cardiovascular diseases; AMI, acute myocardial infarction; TAVR, transcatheter aortic valve replacement; CVD, cerebrovascular disorder; TBI, traumatic brain injury; AKI, acute kidney injury; AF, atrial fibrillation; ACS, acute coronary syndrome; SAP, severe acute pancreatitis; HF, heart failure; CGMS, continuous glucose monitoring system; SMBG, self-monitoring of blood glucose; Lab FPG, laboratory fasting plasma glucose; POCT, point-of-care testing; NA, not available, IHM, in-hospital mortality.

### Meta-analysis results

The associations of GV with the clinical outcomes among the critically ill population were assessed through a meta-analysis, including the 30-day, 90-day, and 1-year ACM, ICU mortality, IHM, and MACE. The results are provided in **Table 2**.

**Table 2:** Meta-analysis results of the glycemic variability (GV) parameters and prognosis in intensive care unit (ICU) patients.

Outcome	No. of studies	Sample size	Heterogeneity		Effect model	HR (95%CI)	*p*
			*I*^2^ (%)	*p*			
30-day ACM	**14**	**65,131**					
CV_con_	7 [12–14,18, 21, 25, 26]	30,541	66.8	0.006	Random	1.22 (1.10–1.36)	<0.001
CV_cat_	9 [6, 8, 13, 18, 20, 22, 23, 25, 35]	36,145	94.2	<0.001	Random	1.35 (1.16–1.56)	<0.001
MAGE	3[9, 21, 22]	21,353	0.00	0.551	Fixed	1.50 (1.27–1.78)	<0.001
90-day ACM	**7**	**16,760**					
CV_con_	5 [13, 15, 16, 18, 26]	14,362	60.6	0.038	Random	1.22 (1.09–1.38)	0.001
CV_cat_	6 [13, 15, 16, 18, 23, 31]	7,771	93.7	<0.001	Random	1.25 (1.07–1.45)	0.005
1-year ACM	**6**	**23,877**					
CV_con_	6 [3, 14–16, 18, 26]	23,877	56.6	0.042	Random	1.19 (1.10–1.30)	<0.001
CV_cat_	4 [3, 15, 16, 18]	11,546	86.3	<0.001	Random	1.12 (1.02–1.23)	0.012
ICU mortality	**5**	**18,208**					
CV_con_	5 [18, 23, 27, 33, 37]	18,208	95.3	<0.001	Random	1.13 (1.06–1.21)	<0.001
IHM	**10**	**39,503**					
CV_con_	4 [3, 18, 19, 24]	14,267	74.6	0.008	Random	1.58 (1.22–2.04)	<0.001
CV_cat_	7 [9, 17, 18, 23, 36, 37]	32,326	95.6	<0.001	Random	1.29 (1.14–1.47)	<0.001
MAGE	3 [9, 17, 36]	15,178	97.1	<0.001	Random	1.16 (1.00–1.33)	0.046
MACE	**6**	**9,065**					
SD	3 [6, 28, 32]	7,855	40.1	0.188	Fixed	2.45 (2.01–2.98)	<0.001
MAGE	3 [29, 34, 38]	1,210	0.00	0.550	Fixed	2.12 (1.44–3.12)	<0.001
DM	**10**						
CV	10 [3, 8, 12–14, 20, 21, 24, 25, 35]	35,430	77.0	<0.001	Random	1.32 (1.13–1.54)	0.001
NDM	**8**						
CV	8 [3, 8, 13, 14, 20, 24, 25, 35]	39,191	88.8	<0.001	Random	1.40 (1.16–1.68)	<0.001

ACM, all-cause mortality; CV_con_, coefficient of variation (continuous); CV_cat_, coefficient of variation (categorical); IHM, in-house mortality; MAGE, mean amplitude of glycemic excursions; MACE, major adverse cardiovascular event; SD, standard deviation; DM, diabetic patients; NDM, non-diabetic patients.

Bold values indicate the number of studies included in each category of analysis.

### 30-day ACM

There were 14 studies (6, 8, 9, 13–15, 19, 21–24, 26, 27, 36) on 65,131 patients that analyzed the effects of CV and MAGE on 30-day ACM. A total of seven studies (13–15, 19, 22, 26, 27) reporting CV_cat_ were pooled. As significant heterogeneity was observed (*I*^2^ = 66.8%, *p* = 0.006), a REM was applied. Among 30,541 patients, the highest CV cohort had a significantly increased risk of 30-day ACM compared with the controls (HR = 1.22, 95%CI = 1.10–1.36, *p* < 0.001). In addition, nine studies (6, 8, 14, 19, 21, 23, 24, 26, 36) reporting CV_con_ were pooled. Significant heterogeneity was also noted (*I*^2^ = 93.6%, *p* < 0.001). The sensitivity analysis suggested that the study by Hou et al. (19) contributed substantially to the notable heterogeneity. After excluding this study, eight studies remained. However, as heterogeneity remained high (*I*^2^ = 94.2%, *p* < 0.001), a REM was used. Among 36,145 patients, each unit of increase in CV_con_ (as a continuous variable) also displayed a notably greater 30-day ACM risk (HR = 1.33, 95%CI = 1.15–1.53, *p* < 0.001).

**Figure 2:**
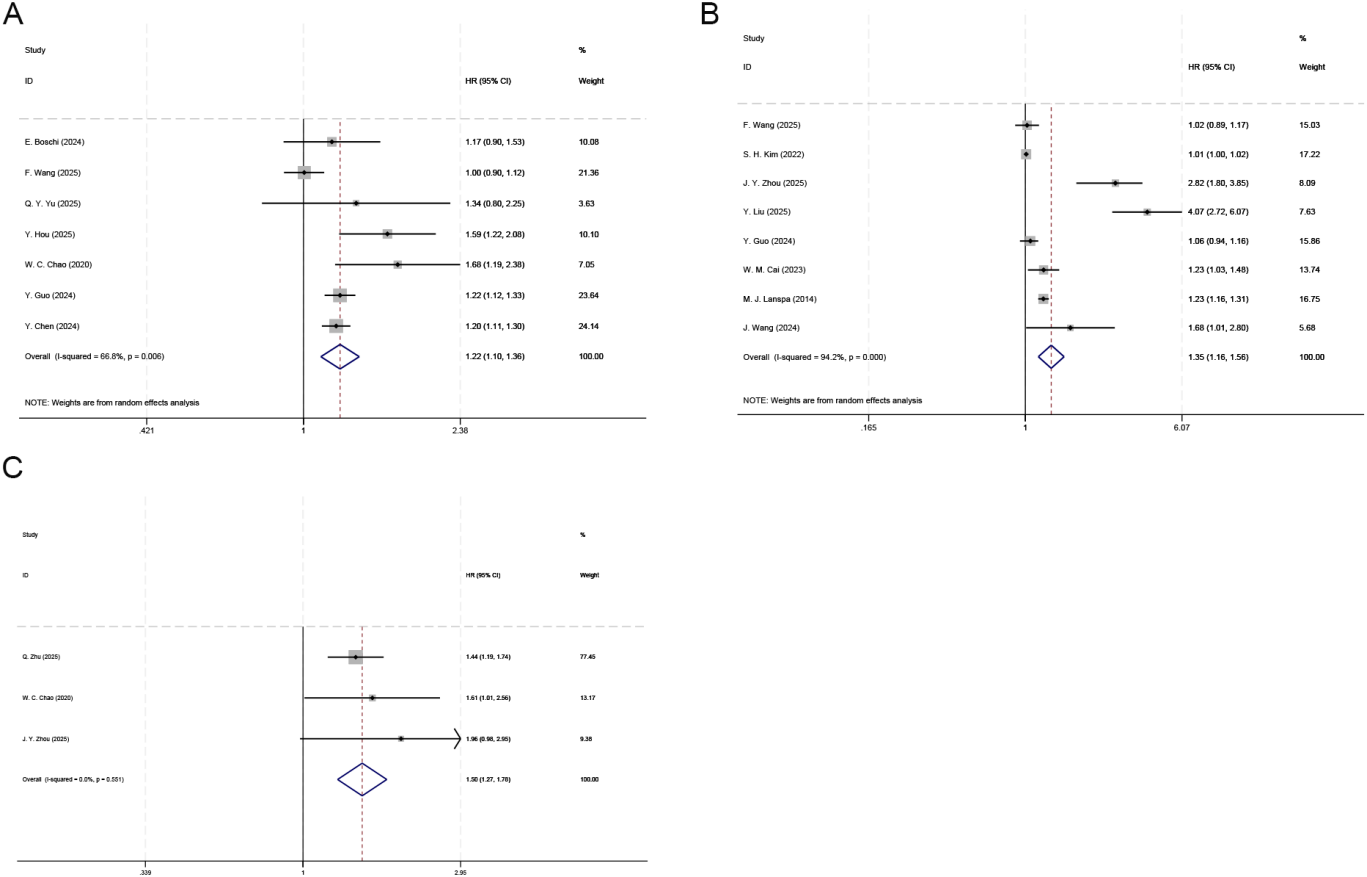
Forest plot of the association between glycemic variability (GV) and 30-day all-cause mortality (ACM) in the critically ill population. **(A)** Categorical coefficient of variation (CV_cat_). **(B)** Continuous CV (CV_con_). **(C)** Mean amplitude of glycemic excursions (MAGE).

Moreover, three studies (9, 22, 23) assessed the association of MAGE with 30-day ACM. Given the low heterogeneity (*I*^2^ = 0.0%, *p* = 0.551), a FEM was applied. Higher MAGE levels were significantly associated with an increased risk of 30-day ACM (HR = 1.50, 95%CI = 1.27–1.78, *p* < 0.001). The results are presented in **Figure 2**.

### In-hospital mortality

There were 10 studies (3, 9, 18–20, 24, 25, 31, 37, 38) on 39,503 patients that reported IHM.

A total of four studies (3, 19, 20, 25) reporting CV_cat_ were pooled. As the heterogeneity was significant (*I*^2^ = 74.6%, *p* = 0.008), a REM was applied. The pooled results showed that the highest CV cohort demonstrated a markedly elevated IHM risk (HR = 1.58, 95%CI = 1.22–2.04, *p* < 0.001). A total of seven studies (9, 18, 19, 24, 31, 37, 38) reporting CV_con_ were pooled. Substantial heterogeneity was noted (*I*^2^ = 95.1%, *p* < 0.001). The sensitivity analysis (**Figure 1**) suggested that the study by Lu et al. (31) contributed substantially to heterogeneity. After this study was excluded, six studies remained. Nevertheless, the heterogeneity remained high (*I*^2^ = 95.6%, *p* < 0.001); therefore, a REM was used. The analysis showed that elevated CV_con_ levels were significantly associated with a higher risk of IHM (HR = 1.29, 95%CI = 1.14–1.47, *p* < 0.001).

There were three studies (9, 18, 37) that evaluated the association between MAGE and IHM. As substantial heterogeneity was observed (*I*^2^ = 97.1%, *p* < 0.001), a REM was applied. Higher MAGE levels were associated with an increased risk of IHM (HR = 1.16, 95%CI = 1.00–1.33, *p* = 0.046). The results are shown in **Supplementary Figure 1**, Document 3, of the **Supplementary Material**.

### 90-day ACM

There were seven studies (14, 16, 17, 19, 24, 27, 32) on 16,760 patients that evaluated 90-day ACM.

A total of five studies (14, 16, 17, 19, 27) reporting CV_cat_ were pooled. As the heterogeneity was substantial (*I*^2^ = 60.6%, *p* = 0.038), a REM was used. The highest CV cohort displayed a notably elevated 90-day ACM risk (HR = 1.22, 95%CI = 1.09–1.38, *p* = 0.001).

A total of six studies (14, 16, 17, 19, 24, 32) reporting CV_con_ were pooled. Due to the significant heterogeneity (*I*^2^ = 93.7%, *p* < 0.001), a REM was applied. Each increment in CV_con_ was significantly associated with an elevated risk of 90-day ACM (HR = 1.25, 95%CI = 1.07–1.45, *p* = 0.005). The results are provided in **Supplementary Figure 2**, Document 3, of the **Supplementary Material**.

### One-year ACM

There were six studies (3, 15–17, 19, 27) on 23,877 patients that analyzed the 1-year ACM. All six studies reported the CV_cat_. As the heterogeneity was moderate (*I*^2^ = 56.6%, *p* = 0.042), a REM was therefore applied. The highest CV cohort exhibited a markedly higher 1-year ACM risk (HR = 1.19, 95%CI = 1.10–1.30, *p* < 0.001).

Moreover, four studies (3, 16, 17, 19) reporting CV_con_ were pooled. Due to the substantial heterogeneity (*I*^2^ = 86.3%, *p* < 0.001), a REM was used. Therefore, each unit of increase in CV was significantly related to an increased 1-year ACM risk (HR = 1.12, 95%CI = 1.02–1.23, *p* = 0.012). The results are presented in **Supplementary Figure 3**, Document 3, of the **Supplementary Material**.

### ICU mortality

There were five studies (19, 24, 28, 34, 38) on 18,208 patients that evaluated ICU mortality.

All five studies reported CV_con_. As the heterogeneity was significant (*I*^2^ = 95.3%, *p* < 0.001), a REM was applied. Each unit of increase in CV_con_ was associated with a significantly elevated risk of ICU mortality (HR = 1.13, 95%CI = 1.06–1.21, *p* < 0.001). The results are provided in **Supplementary Figure 4**, Document 3, of the **Supplementary Material**.

### Major adverse cardiovascular event

There were six studies (6, 29, 30, 33, 35, 39) on 9,065 patients that analyzed MACE.

A total of three studies reporting SD (6, 29, 33) were pooled. Due to the heterogeneity being low (*I*^2^ = 40.1%, *p* = 0.188), a FEM was applied. The high SD cohort demonstrated a notably higher MACE risk than the controls (HR = 2.45, 95%CI = 2.01–2.98, *p* < 0.001), demonstrating strong statistical significance.

In addition, three studies reporting MAGE (30, 35, 39) were pooled. Given the insignificant heterogeneity (*I*^2^ = 0.0%, *p* = 0.550), a FEM was applied. The high MAGE cohort had a markedly increased MACE risk relative to the controls (HR = 2.12, 95%CI = 1.44–3.12, *p* < 0.001). The results are shown in **Supplementary Figure 5**, Document 3, of the **Supplementary Material**.

### Subgroup analysis based on diabetes status

There were 10 studies (3, 8, 13–15, 21, 22, 25, 26, 36) on 35,430 DM patients that reported relevant outcomes. The studies reporting CV were pooled. As the heterogeneity was substantial (*I*^2^ = 77%, *p* < 0.001), a REM was used. DM patients with elevated CV levels displayed a notably higher 30-day ACM risk than the control cohort (HR = 1.32, 95%CI = 1.13–1.54, *p* = 0.001).

In addition, eight studies on 34,739 NDM patients (3, 8, 14, 15, 21, 25, 26, 36) were analyzed. As the heterogeneity was considerable (*I*^2^ = 88.8%, *p* < 0.001), a REM was applied. The NDM cohort with increased CV variability displayed a markedly higher 30-day ACM risk than the control cohort (HR = 1.40, 95%CI = 1.16–1.68, *p* < 0.001). The results are provided in **Supplementary Figure 6**, Document 3, of the **Supplementary Material**.

### Subgroup analysis for 30-day ACM stratified by disease category

To address these clinical constraints and investigate residual heterogeneity, an exploratory subgroup analysis for 30-day ACM was performed by classifying patients into three primary critical illness categories: cardiovascular (6, 14) (*n* = 2), sepsis (23) (*n* = 1), and neurological patients (8, 24) (*n* = 2). In this subgroup analysis, five studies that uniformly modeled GV as a continuous metric (CV_con_) were included. A REM was used. The subgroup analysis results revealed that the prognostic strength of continuous GV was substantially altered by the underlying diagnosis. In the sepsis subgroup, represented by a single study, a higher CV_con_ was significantly associated with an increased risk of short-term mortality (HR = 2.82, 95%CI = 1.80–3.85, *p* < 0.001). Conversely, the associations did not achieve statistical significance in the remaining two categories. In neurological patients, CV_con_ demonstrated a positive trend, but failed to reach statistical significance (subtotal HR = 2.21, 95%CI = 0.69–7.11, *p* = 0.181), with significant heterogeneity (*I*^2^ = 96.5%, *p* < 0.001). Similarly, for cardiovascular patients, no significant association was observed (subtotal HR = 1.23, 95%CI = 0.77–1.97, *p* = 0.387; *I*^2^ = 70.9%, *p* = 0.064). The overall pooled estimate for all five studies on CV_con_ remained significant (overall HR = 1.84, 95%CI = 1.17–2.89, *p* < 0.001; *I*^2^ = 93.4%). The results are provided in Document 3, **Supplementary Figure 7**, of the **Supplementary Material**.

### Sensitivity analysis results

The influence of individual studies on the overall results was evaluated using leave-one-out sensitivity analyses for the 30-day, 90-day, and 1-year ACM, ICU mortality, IHM, and MACE.

Except for the 30-day ACM (CV_con_) and the IHM (CV_con_), the sensitivity analyses demonstrated that the direction and magnitude of the pooled effect estimates for 30-day ACM (CV_cat_), 90-day ACM, 1-year ACM, and MACE remained largely unchanged after the exclusion of the studies that contributed substantial heterogeneity, indicating good stability of the findings. Overall, these results support the reliability and robustness of the meta-analysis. Detailed sensitivity analysis results are provided in Document 3 of the **Supplementary Material**.

### Publication bias

**Figure 3:**
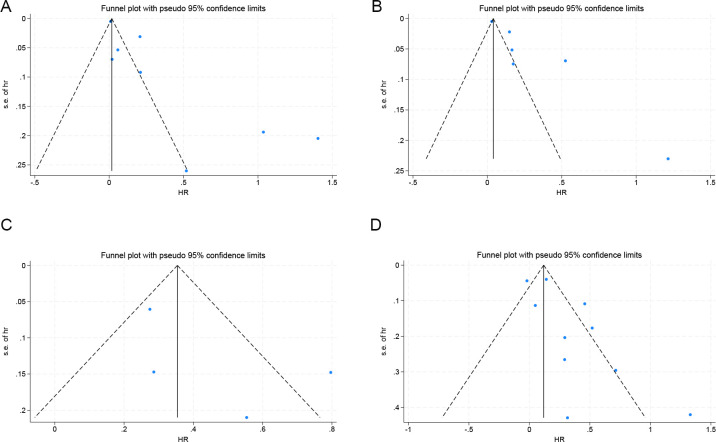
Funnel plots for publication bias. **(A)** The 30-day all-cause mortality (ACM) (continuous coefficient of variation, CV_con_). **(B)** In-hospital mortality (IHM) (CV_con_). **(C)** IHM (categorical CV, CV_cat_). **(D)** The 30-day ACM in diabetic (DM) patients (CV).

For outcomes including more than 10 studies, funnel plots and Egger’s test were used to assess publication bias and evaluate the reliability of the pooled estimates. The funnel plots for 30-day ACM (CV_con_) and IHM (both CV_con_ and CV_cat_) demonstrated noticeable asymmetry, while Egger’s test suggested potential publication bias (*p* = 0.019, 0.012, and 0.008, respectively). Detailed results are shown in **Figure 3**.

## Discussion

This meta-analysis demonstrated that elevated GV, particularly MAGE and CV, independently predicts both short- and long-term ACM and MACE in critically ill patients. Compared with SD and CV, MAGE may provide superior prognostic value for mortality risk assessment.

Among the evaluated parameters, MAGE exhibited the largest effect size for predicting short-term mortality (HR = 1.50). Its advantage may stem from its ability to more accurately capture extreme glucose excursions and glycemic instability, which aligns with previous findings (35, 40). However, although MAGE showed a larger pooled effect size, this observation should be interpreted with caution. No formal head-to-head statistical comparison between the GV metrics was conducted, and the relatively small number of studies evaluating MAGE (*n* = 3), compared with CV (*n* = 14), may have limited the stability of this estimate. In contrast, CV minimizes the influence of the baseline mean glucose levels and therefore demonstrated relatively stable predictive performance in both the DM and NDM subgroups, with an approximately 30% increase in risk for each logarithmic unit increase. Nevertheless, CV may be affected by factors such as baseline patient characteristics and clinical interventions, potentially limiting its predictive performance in certain settings (36). Subgroup analyses further indicated that NDM patients may have lower tolerance to glycemic fluctuations, possibly due to the metabolic “preconditioning” developed in DM patients following chronic exposure to hyperglycemia (4, 41).

Our findings suggest that glycemic management in critically ill patients should focus not only on controlling the mean glucose levels but also on minimizing glucose fluctuations. Indicators such as MAGE may therefore be valuable for outcome prediction and for guiding interventions aimed at improving prognosis (40). Moreover, our finding that elevated SD (HR = 2.45) and MAGE (HR = 2.12) were strongly associated with an increased MACE risk is consistent with the recent report by Darouei et al. (10). Furthermore, elevated GV was significantly associated with short-term ACM and long-term adverse outcomes, including MACE and cognitive decline (7, 42).

Based on these findings, effective control of GV appears crucial for improving the clinical outcomes of critically ill patients. Individuals with marked glucose fluctuations may require more precise and individualized glycemic management strategies. Reducing GV has been shown to significantly decrease IHM and the incidence of major complications (6, 35). Therefore, critical care glycemic management has evolved from a sole focus on “mean glucose control” to “variability management.” Several key strategies have been proposed.

The first involves technology-driven strategies. Multiple studies support the use of continuous glucose monitoring (CGM) to overcome the limitations of traditional point-of-care testing (POCT), which may fail to detect more than 30% of hypoglycemic episodes and rapid glucose fluctuations (43, 44). Both the 2025 American Diabetes Association (ADA) guidelines (45) and the 2024 Society of Critical Care Medicine (SCCM) expert consensus (46) emphasize that CGM provides real-time trends in glucose variability, enabling healthcare providers to intervene proactively before the glucose levels exceed the warning thresholds. The second approach is protocol-driven management. Evidence suggests that structured, algorithm-based, nurse-led insulin protocols, such as the Space GlucoseControl (SGC) system, can significantly shorten the time required for blood glucose to return to target ranges and reduce the glycemic fluctuations caused by delays in physician orders (47–49). The third approach involves synchronized nutritional management. Interventions targeting GV should not rely solely on pharmacological therapy, as dietary regulation and optimized nutritional support also play essential roles in maintaining glucose stability (34). Continuous infusion of low-glycemic index (GI) formulas combined with dynamic monitoring of gastric residual volume may help reduce the iatrogenic glucose fluctuations resulting from interruptions in nutritional delivery. Overall, multidisciplinary collaboration is essential in the management of critically ill patients to develop individualized therapeutic strategies and maximize improvements in patient prognosis.

The present study synthesized the currently available evidence on multiple GV parameters from nearly 100,000 patients through a comprehensive literature search. Both time domain (SD and CV) and amplitude domain (MAGE) indicators were evaluated, providing high-quality evidence to inform future clinical guideline development. However, there are a number of limitations. Firstly, there was substantial heterogeneity among the included cohorts due to differences in the glucose monitoring methods, which introduced considerable technical variability. Intermittent sampling methods, such as POCT or the blood gas measurements performed at frequencies ranging from hourly to twice daily, inherently tend to underestimate true peak-to-trough glucose excursions compared with CGM. This methodological variability likely contributed substantially to the high statistical heterogeneity (*I*^2^ frequently >90%) in the continuous metric analyses. Secondly, the definitions of outcome measures such as MACE were not entirely consistent across studies. Thirdly, meta-analyses cannot fully eliminate the potential confounding effects of vasoactive agents and corticosteroid use, both of which may influence GV. The publication bias was significant for certain outcomes, particularly IHM (for both CV_con_ and CV_cat_). To address this issue, trim-and-fill analyses were conducted, which demonstrated that the adjusted HRs remained statistically significant, supporting the overall robustness of the primary findings despite the presence of publication bias. Moreover, our exploratory disease-specific subgroup analysis for 30-day ACM was constrained by a small number of eligible studies (*n* = 5). The sepsis subgroup contained only one study and the remaining subgroups contained only two studies each, which expanded the CIs and reduced the statistical power. Therefore, these findings should still be interpreted with caution in clinical practice. Future studies should standardize the glucose monitoring protocols, harmonize the outcome definitions, and rigorously control for confounding factors to further improve the clinical applicability of the GV parameters in precision medicine.

## Conclusion

Dynamic monitoring of GV should be emphasized in clinical practice, particularly for MAGE and CV. Future studies should standardize the assessment of GV metrics and further clarify the relationship between glucose fluctuations and other complications. Moreover, integrating artificial intelligence with multimodal physiological signals may facilitate the development of real-time early warning models for GV. Future randomized controlled trials are also needed to evaluate the effectiveness of interventions targeting glycemic fluctuations, particularly across different diabetes statuses, and to determine how specific intervention strategies influence GV control and improve clinical outcomes.

## Data Availability

The original contributions presented in the study are included in the article/**Supplementary Material**. Further inquiries can be directed to the corresponding author.
