# Supplementary material for: Predictive value of different glycemic variability indicators for prognosis in critically ill patients: a meta-analysis
Source: Front Endocrinol (Lausanne). 2026 Jun 22;17:1857523. doi: 10.3389/fendo.2026.1857523 (PMC13333409; doi:10.3389/fendo.2026.1857523)
Supplement: Supplementary file 1 [file DataSheet1.docx]

Supplementary document 1 Search strategy

**The search strategy (Pubmed)**

| Search number | Query | Results |
| --- | --- | --- |
| 1 | (Critical Illness[MeSH Terms]) OR (Sepsis[MeSH Terms])) OR (Acute Coronary Syndrome[MeSH Terms])) OR (Multiple Organ Failure[MeSH Terms])) OR (Respiratory Distress Syndrome[MeSH Terms])) OR (Multiple Trauma[MeSH Terms])) OR (Shock[MeSH Terms])) OR (Acute Kidney Injury[MeSH Terms]) | 374,503 |
| 2 | "abdominal sepsis"[Title/Abstract] OR "acute coronary syndrome"[Title/Abstract] OR "acute coronary syndromes"[Title/Abstract] OR "acute kidney failure"[Title/Abstract] OR "Acute Kidney Failures"[Title/Abstract] OR "Acute Kidney Injuries"[Title/Abstract] OR "acute kidney injury"[Title/Abstract] OR "Acute Kidney Insufficiencies"[Title/Abstract] OR "acute kidney insufficiency"[Title/Abstract] OR "acute renal failure"[Title/Abstract] OR "Acute Renal Failures"[Title/Abstract] OR "Acute Renal Injuries"[Title/Abstract] OR "Acute Renal Injury"[Title/Abstract] OR "Acute Renal Insufficiencies"[Title/Abstract] OR "acute renal insufficiency"[Title/Abstract] OR "Acute Respiratory Distress Syndrome"[Title/Abstract] OR "Adult Respiratory Distress Syndrome"[Title/Abstract] OR "Blood Poisoning"[Title/Abstract] OR "Blood Poisonings"[Title/Abstract] OR "Bloodstream Infection"[Title/Abstract] OR "Bloodstream Infections"[Title/Abstract] OR "breathing distress syndrome"[Title/Abstract] OR "cardiovascular collapse"[Title/Abstract] OR "circulation shock"[Title/Abstract] OR "circulatory collapse"[Title/Abstract] OR "Circulatory Failure"[Title/Abstract] OR "circulatory shock"[Title/Abstract] OR "critical illness"[Title/Abstract] OR "Critical Illnesses"[Title/Abstract] OR "Critically Ill"[Title/Abstract] OR "focal sepsis"[Title/Abstract] OR "Human ARDS"[Title/Abstract] OR "Hypovolemic Shock"[Title/Abstract] OR "incremental shock"[Title/Abstract] OR "intraabdominal sepsis"[Title/Abstract] OR "kidney acute failure"[Title/Abstract] OR "lung distress syndrome"[Title/Abstract] OR "massive trauma"[Title/Abstract] OR "MODS"[Title/Abstract] OR "MODS multiple organ dysfunction syndrome"[Title/Abstract] OR "multi injury"[Title/Abstract] OR "multi organ dysfunction syndrome"[Title/Abstract] OR "multi trauma"[Title/Abstract] OR "multiinjury"[Title/Abstract] OR "multi-organ disease"[Title/Abstract] OR "multiorgan dysfunction syndrome"[Title/Abstract] OR "multiorgan failure"[Title/Abstract] OR "Multiple Injur*"[Title/Abstract] OR "multiple organ disease"[Title/Abstract] OR "Multiple Organ Dysfunction Syndrome"[Title/Abstract] OR "Multiple Organ Failur*"[Title/Abstract] OR "Multiple Trauma"[Title/Abstract] OR "Multiple Traumas"[Title/Abstract] OR "Multiple Wound"[Title/Abstract] OR "Multiple Wounds"[Title/Abstract] OR "multisystem organ failure"[Title/Abstract] OR "multitrauma"[Title/Abstract] OR "nonseptic shock"[Title/Abstract] OR "Pediatric Respiratory Distress Syndrome"[Title/Abstract] OR "polyinjured patient"[Title/Abstract] OR "poly-injuries"[Title/Abstract] OR "polytraumatized patient"[Title/Abstract] OR "pulmonary distress syndrome"[Title/Abstract] OR "Pyaemias"[Title/Abstract] OR "Pyemia*"[Title/Abstract] OR "Pyohemia*"[Title/Abstract] OR "RDS"[Title/Abstract] OR "respiration distress syndrome"[Title/Abstract] OR "Respiratory Distress Syndromes"[Title/Abstract] OR "Sepsis"[Title/Abstract] OR "sepsis syndrome"[Title/Abstract] OR "septic disease"[Title/Abstract] OR "Septicemia"[Title/Abstract] OR "Septicemias"[Title/Abstract] OR "Severe Sepsis"[Title/Abstract] OR "Shock"[Title/Abstract] OR "shock index"[Title/Abstract] OR "shock intensity"[Title/Abstract] OR "Shock Lung"[Title/Abstract] OR "shock syndrome"[Title/Abstract] OR "surgical shock"[Title/Abstract] | 594,887 |
| 3 | Blood Glucose[MeSH Terms] | 193,535 |
| 4 | "blood glucose"[Title/Abstract] OR "blood glucose level"[Title/Abstract] OR "blood serum glucose"[Title/Abstract] OR "blood sugar"[Title/Abstract] OR "blood sugar level"[Title/Abstract] OR "glucosaemia"[Title/Abstract] OR "glucose blood level"[Title/Abstract] OR "Glucose Clamp Technique"[Title/Abstract] OR "Glucose Tolerance Test"[Title/Abstract] OR "glucosemia"[Title/Abstract] OR "glycaemia"[Title/Abstract] OR "Glycated Hemoglobin"[Title/Abstract] OR "glycemia"[Title/Abstract] OR "Glycemic Index"[Title/Abstract] OR "Hyperglycemia"[Title/Abstract] OR "Hypoglycemia"[Title/Abstract] OR "normoglycaemia"[Title/Abstract] OR "normoglycemia"[Title/Abstract] OR "plasma glucose"[Title/Abstract] OR "postprandial glycaemia"[Title/Abstract] OR "postprandial glycemia"[Title/Abstract] OR "serum glucose"[Title/Abstract] OR "serum sugar"[Title/Abstract] | 265,288 |
| 5 | varia*[Title/Abstract] | 2,932,568 |
| 6 | (#1 OR #2 ) AND (#3 OR #4 ) AND #5 | 1279 |

**The search strategy (Embase)**

| Search number | Query | Results |
| --- | --- | --- |
| 1 | 'critical illness'/exp OR 'sepsis'/exp OR 'acute coronary syndrome'/exp OR 'multiple organ failure'/exp OR 'respiratory distress syndrome'/exp OR 'multiple trauma'/exp OR 'shock'/exp OR 'acute kidney injury'/exp | 877213 |
| 2 | 'abdominal sepsis':ti,ab,kw OR 'acute coronary syndrome':ti,ab,kw OR 'acute coronary syndromes':ti,ab,kw OR 'acute kidney failure':ti,ab,kw OR 'acute kidney failures':ti,ab,kw OR 'acute kidney injuries':ti,ab,kw OR 'acute kidney injury':ti,ab,kw OR 'acute kidney insufficiencies':ti,ab,kw OR 'acute kidney insufficiency':ti,ab,kw OR 'acute renal failure':ti,ab,kw OR 'acute renal failures':ti,ab,kw OR 'acute renal injuries':ti,ab,kw OR 'acute renal injury':ti,ab,kw OR 'acute renal insufficiencies':ti,ab,kw OR 'acute renal insufficiency':ti,ab,kw OR 'acute respiratory distress syndrome':ti,ab,kw OR 'adult respiratory distress syndrome':ti,ab,kw OR 'blood poisoning':ti,ab,kw OR 'blood poisonings':ti,ab,kw OR 'bloodstream infection':ti,ab,kw OR 'bloodstream infections':ti,ab,kw OR 'breathing distress syndrome':ti,ab,kw OR 'mental shock':ti,ab,kw OR 'intraabdominal sepsis':ti,ab,kw OR 'kidney acute failure':ti,ab,kw OR 'lung distress syndrome':ti,ab,kw OR 'massive trauma':ti,ab,kw OR 'mods':ti,ab,kw OR 'mods multiple organ dysfunction syndrome':ti,ab,kw OR 'multi injury':ti,ab,kw OR 'multi organ dysfunction syndrome':ti,ab,kw OR 'multi trauma':ti,ab,kw OR 'multiinjury':ti,ab,kw OR 'multi-organ disease':ti,ab,kw OR 'multiorgan dysfunction syndrome':ti,ab,kw OR 'multiorgan failure':ti,ab,kw OR 'multiple injur*':ti,ab,kw OR 'multiple organ disease':ti,ab,kw OR 'multiple organ dysfunction syndrome':ti,ab,kw OR 'multiple organ failur*':ti,ab,kw OR 'multiple trauma':ti,ab,kw OR 'multiple traumas':ti,ab,kw OR 'multiple wound':ti,ab,kw OR 'multiple wounds':ti,ab,kw OR 'multisystem organ failure':ti,ab,kw OR 'multitrauma':ti,ab,kw OR 'nonseptic shock':ti,ab,kw OR 'pediatric respiratory distress syndrome':ti,ab,kw OR 'polyinjured patient':ti,ab,kw OR 'poly-injuries':ti,ab,kw OR 'polytraumatized patient':ti,ab,kw OR 'pulmonary distress syndrome':ti,ab,kw OR 'pyaemias':ti,ab,kw OR 'pyemia*':ti,ab,kw OR 'pyohemia*':ti,ab,kw OR 'rds':ti,ab,kw OR 'respiration distress syndrome':ti,ab,kw OR 'respiratory distress syndromes':ti,ab,kw OR 'sepsis':ti,ab,kw OR 'sepsis syndrome':ti,ab,kw OR 'septic disease':ti,ab,kw OR 'septicemia':ti,ab,kw OR 'septicemias':ti,ab,kw OR 'severe sepsis':ti,ab,kw OR 'shock':ti,ab,kw OR 'shock index':ti,ab,kw OR 'shock intensity':ti,ab,kw OR 'shock lung':ti,ab,kw OR 'shock syndrome':ti,ab,kw OR 'surgical shock':ti,ab,kw | 792368 |
| 3 | 'glucose blood level'/exp | 391449 |
| 4 | 'blood glucose':ti,ab,kw OR 'blood glucose level':ti,ab,kw OR 'blood serum glucose':ti,ab,kw OR 'blood sugar':ti,ab,kw OR 'blood sugar level':ti,ab,kw OR 'glucosaemia':ti,ab,kw OR 'glucose blood level':ti,ab,kw OR 'glucose clamp technique':ti,ab,kw OR 'glucose tolerance test':ti,ab,kw OR 'glucosemia':ti,ab,kw OR 'glycaemia':ti,ab,kw OR 'glycated hemoglobin':ti,ab,kw OR 'glycemia':ti,ab,kw OR 'glycemic index':ti,ab,kw OR 'hyperglycemia':ti,ab,kw OR 'hypoglycemia':ti,ab,kw OR 'normoglycaemia':ti,ab,kw OR 'normoglycemia':ti,ab,kw OR 'plasma glucose':ti,ab,kw OR 'postprandial glycaemia':ti,ab,kw OR 'postprandial glycemia':ti,ab,kw OR 'serum glucose':ti,ab,kw OR 'serum sugar':ti,ab,kw | 409860 |
| 5 | varia* | 4310219 |
| 6 | (#1 OR #2) AND (#3 OR #4) AND #5 | 2574 |

**The search strategy (Web of Science)**

| Search number | Query | Results |
| --- | --- | --- |
| 1 | (abdominal sepsis) OR (acute coronary syndrome) OR (acute coronary syndromes) OR (acute kidney failure) OR (Acute Kidney Failures) OR (Acute Kidney Injuries) OR (acute kidney injury) OR (Acute Kidney Insufficiencies) OR (acute kidney insufficiency) OR (acute renal failure) OR (Acute Renal Failures) OR (Acute Renal Injuries) OR (Acute Renal Injury) OR (Acute Renal Insufficiencies) OR (acute renal insufficiency) OR (Acute Respiratory Distress Syndrome) OR (Adult Respiratory Distress Syndrome) OR (Blood Poisoning) OR (Blood Poisonings) OR (Bloodstream Infection) OR (Bloodstream Infections) OR (breathing distress syndrome) OR (cardiovascular collapse) OR (circulation shock) OR (circulatory collapse) OR (Circulatory Failure) OR (circulatory shock) OR (critical illness) OR (Critical Illnesses) OR (Critically Ill) OR (focal sepsis) OR (Human ARDS) OR (Hypovolemic Shock) OR (incremental shock) OR (intraabdominal sepsis) OR (kidney acute failure) OR (lung distress syndrome) OR (massive trauma) OR (MODS) OR (MODS multiple organ dysfunction syndrome) OR (multi injury) OR (multi organ dysfunction syndrome) OR (multi trauma) OR (multiinjury) OR (multi-organ disease) OR (multiorgan dysfunction syndrome) OR (multiorgan failure) OR (Multiple Injur*) OR (multiple organ disease) OR (Multiple Organ Dysfunction Syndrome) OR (Multiple Organ Failur*) OR (Multiple Trauma) OR (Multiple Traumas) OR (Multiple Wound) OR (Multiple Wounds) OR (multisystem organ failure) OR (multitrauma) OR (nonseptic shock) OR (Pediatric Respiratory Distress Syndrome) OR (polyinjured patient) OR (poly-injuries) OR (polytraumatized patient) OR (pulmonary distress syndrome) OR (Pyaemias) OR (Pyemia*) OR (Pyohemia*) OR (RDS) OR (respiration distress syndrome) OR (Respiratory Distress Syndromes) OR (Sepsis) OR (sepsis syndrome) OR (septic disease) OR (Septicemia) OR (Septicemias) OR (Severe Sepsis) OR (Shock) OR (shock index) OR (shock intensity) OR (Shock Lung) OR (shock syndrome) OR (surgical shock) (Topic) | 1203446 |
| 2 | TS=((blood glucose) OR (blood glucose level) OR (blood serum glucose) OR (blood sugar) OR (blood sugar level) OR (glucosaemia) OR (glucose blood level) OR (Glucose Clamp Technique) OR (Glucose Tolerance Test) OR (glucosemia) OR (glycaemia) OR (Glycated Hemoglobin) OR (glycemia) OR (Glycemic Index) OR (Hyperglycemia) OR (Hypoglycemia) OR (normoglycaemia) OR (normoglycemia) OR (plasma glucose) OR (postprandial glycaemia) OR (postprandial glycemia) OR (serum glucose) OR (serum sugar) ) | 466355 |
| 3 | TS=((varia*)) | 6608573 |
| 4 | #3 AND #2 AND #1 | 2379 |

**The search strategy (Cochrane Library)**

| Search number | Query | Results |
| --- | --- | --- |
| 1 | MeSH descriptor: [Critical Illness] explode all trees | 3807 |
| 2 | MeSH descriptor: [Sepsis] explode all trees | 6552 |
| 3 | MeSH descriptor: [Acute Coronary Syndrome] explode all trees | 3121 |
| 4 | MeSH descriptor: [Multiple Organ Failure] explode all trees | 553 |
| 5 | MeSH descriptor: [Respiratory Distress Syndrome] explode all trees | 3612 |
| 6 | MeSH descriptor: [Multiple Trauma] explode all trees | 325 |
| 7 | MeSH descriptor: [Shock] explode all trees | 3528 |
| 8 | MeSH descriptor: [Acute Kidney Injury] explode all trees | 2469 |
| 9 | ('abdominal sepsis' OR 'acute coronary syndrome' OR 'acute coronary syndromes' OR 'acute kidney failure' OR 'Acute Kidney Failures' OR 'Acute Kidney Injuries' OR 'acute kidney injury' OR 'Acute Kidney Insufficiencies' OR 'acute kidney insufficiency' OR 'acute renal failure' OR 'Acute Renal Failures' OR 'Acute Renal Injuries' OR 'Acute Renal Injury' OR 'Acute Renal Insufficiencies' OR 'acute renal insufficiency' OR 'Acute Respiratory Distress Syndrome' OR 'Adult Respiratory Distress Syndrome' OR 'Blood Poisoning' OR 'Blood Poisonings' OR 'Bloodstream Infection' OR 'Bloodstream Infections' OR 'breathing distress syndrome' OR‘mental shock' OR 'intraabdominal sepsis' OR 'kidney acute failure' OR 'lung distress syndrome' OR 'massive trauma' OR 'MODS' OR 'MODS multiple organ dysfunction syndrome' OR 'multi injury' OR 'multi organ dysfunction syndrome' OR 'multi trauma' OR 'multiinjury' OR 'multi-organ disease' OR 'multiorgan dysfunction syndrome' OR 'multiorgan failure' OR 'Multiple Injur*' OR 'multiple organ disease' OR 'Multiple Organ Dysfunction Syndrome' OR 'Multiple Organ Failur*' OR 'Multiple Trauma' OR 'Multiple Traumas' OR 'Multiple Wound' OR 'Multiple Wounds' OR 'multisystem organ failure' OR 'multitrauma' OR 'nonseptic shock' OR 'Pediatric Respiratory Distress Syndrome' OR 'polyinjured patient' OR 'poly-injuries' OR 'polytraumatized patient' OR 'pulmonary distress syndrome' OR 'Pyaemias' OR 'Pyemia*' OR 'Pyohemia*' OR 'RDS' OR 'respiration distress syndrome' OR 'Respiratory Distress Syndromes' OR 'Sepsis' OR 'sepsis syndrome' OR 'septic disease' OR 'Septicemia' OR 'Septicemias' OR 'Severe Sepsis' OR 'Shock' OR 'shock index' OR 'shock intensity' OR 'Shock Lung' OR 'shock syndrome' OR 'surgical shock'):ti,ab,kw | 67041 |
| 10 | MeSH descriptor: [Blood Glucose] explode all trees | 22329 |
| 11 | ('blood glucose' OR 'blood glucose level' OR 'blood serum glucose' OR 'blood sugar' OR 'blood sugar level' OR 'glucosaemia' OR 'glucose blood level' OR 'Glucose Clamp Technique' OR 'Glucose Tolerance Test' OR 'glucosemia' OR 'glycaemia' OR 'Glycated Hemoglobin' OR 'glycemia' OR 'Glycemic Index' OR 'Hyperglycemia' OR 'Hypoglycemia' OR 'normoglycaemia' OR 'normoglycemia' OR 'plasma glucose' OR 'postprandial glycaemia' OR 'postprandial glycemia' OR 'serum glucose' OR 'serum sugar'):ti,ab,kw | 90908 |
| 12 | varia* | 227426 |
| 13 | ( #1 OR #2 OR #3 OR #4 OR #5 OR #6 OR #7 OR #8 OR #9 ) AND ( #10 OR #11 ) and #12 | 486 |

Supplementary document 2 Quality assessment of included studies

| **Author, year** | **selection** | | | | **Comparability** | **Outcome** | | | **Total Score** |
| --- | --- | --- | --- | --- | --- | --- | --- | --- | --- |
|  | **#1** | **#2** | **#3** | **#4** | **#1** | **#1** | **#2** | **#3** |  |
| E. Boschi 2024 | 1 | 1 | 1 | 1 | 1 | 0 | 1 | 1 | 7 |
| F. Wang 2025 | 1 | 1 | 1 | 1 | 2 | 1 | 1 | 1 | 9 |
| J. Wang 2024 | 1 | 1 | 1 | 1 | 2 | 1 | 1 | 1 | 9 |
| Q. Zhu 2025 | 1 | 1 | 1 | 1 | 2 | 1 | 1 | 1 | 9 |
| Q. Y. Yu 2025 | 1 | 1 | 1 | 1 | 2 | 1 | 1 | 1 | 9 |
| H. Yang 2025 | 1 | 1 | 1 | 1 | 2 | 1 | 1 | 1 | 9 |
| D. Wang 2025 | 1 | 1 | 1 | 1 | 2 | 1 | 1 | 1 | 9 |
| W. L. Shuai 2025 | 1 | 1 | 1 | 1 | 1 | 1 | 1 | 1 | 8 |
| P. Prakash 2025 | 1 | 1 | 1 | 1 | 1 | 1 | 1 | 1 | 8 |
| Y. Hou 2025 | 1 | 1 | 1 | 1 | 2 | 1 | 1 | 1 | 9 |
| J. Chen 2024 | 1 | 1 | 1 | 1 | 1 | 1 | 1 | 1 | 8 |
| S. H. Kim 2022 | 1 | 1 | 1 | 1 | 2 | 1 | 1 | 1 | 9 |
| W. C. Chao 2020 | 1 | 1 | 1 | 1 | 2 | 1 | 1 | 1 | 9 |
| J. Y. Zhou 2025 | 1 | 1 | 1 | 1 | 2 | 1 | 1 | 1 | 9 |
| Y. Liu 2025 | 1 | 1 | 1 | 1 | 2 | 1 | 1 | 1 | 9 |
| L. R. Qi 2024 | 1 | 1 | 1 | 1 | 2 | 1 | 1 | 1 | 9 |
| Y. Guo 2024 | 1 | 1 | 1 | 1 | 2 | 1 | 1 | 1 | 9 |
| Y. Chen 2024 | 1 | 1 | 1 | 1 | 2 | 1 | 1 | 0 | 8 |
| W. M. Cai 2022 | 1 | 1 | 1 | 1 | 2 | 1 | 1 | 1 | 9 |
| Z. Lu 2022 | 1 | 1 | 1 | 1 | 2 | 1 | 1 | 1 | 9 |
| E. Gerbaud 2022 | 1 | 1 | 1 | 1 | 1 | 1 | 1 | 1 | 8 |
| G. Su 2021 | 1 | 1 | 1 | 1 | 2 | 1 | 1 | 1 | 9 |
| Y. Lu 2021 | 1 | 1 | 1 | 1 | 1 | 0 | 1 | 0 | 6 |
| Y. Cai 2020 | 1 | 1 | 1 | 0 | 1 | 1 | 1 | 0 | 6 |
| E. Gerbaud 2019 | 1 | 1 | 1 | 1 | 1 | 1 | 1 | 1 | 8 |
| R. Doola 2019 | 1 | 1 | 1 | 1 | 1 | 1 | 1 | 1 | 8 |
| H. Takahashi 2018 | 1 | 1 | 1 | 1 | 2 | 1 | 1 | 0 | 8 |
| M. J. Lanspa 2014 | 1 | 1 | 1 | 1 | 2 | 0 | 1 | 1 | 8 |
| N. A. Ali 2008 | 1 | 1 | 1 | 1 | 1 | 1 | 1 | 1 | 8 |
| M. Egi 2006 | 1 | 1 | 1 | 1 | 2 | 1 | 1 | 1 | 9 |
| X. F. Wang 2014 | 1 | 1 | 1 | 1 | 1 | 1 | 1 | 1 | 8 |

Supplementary document 3 Other analysis results

Forest plot


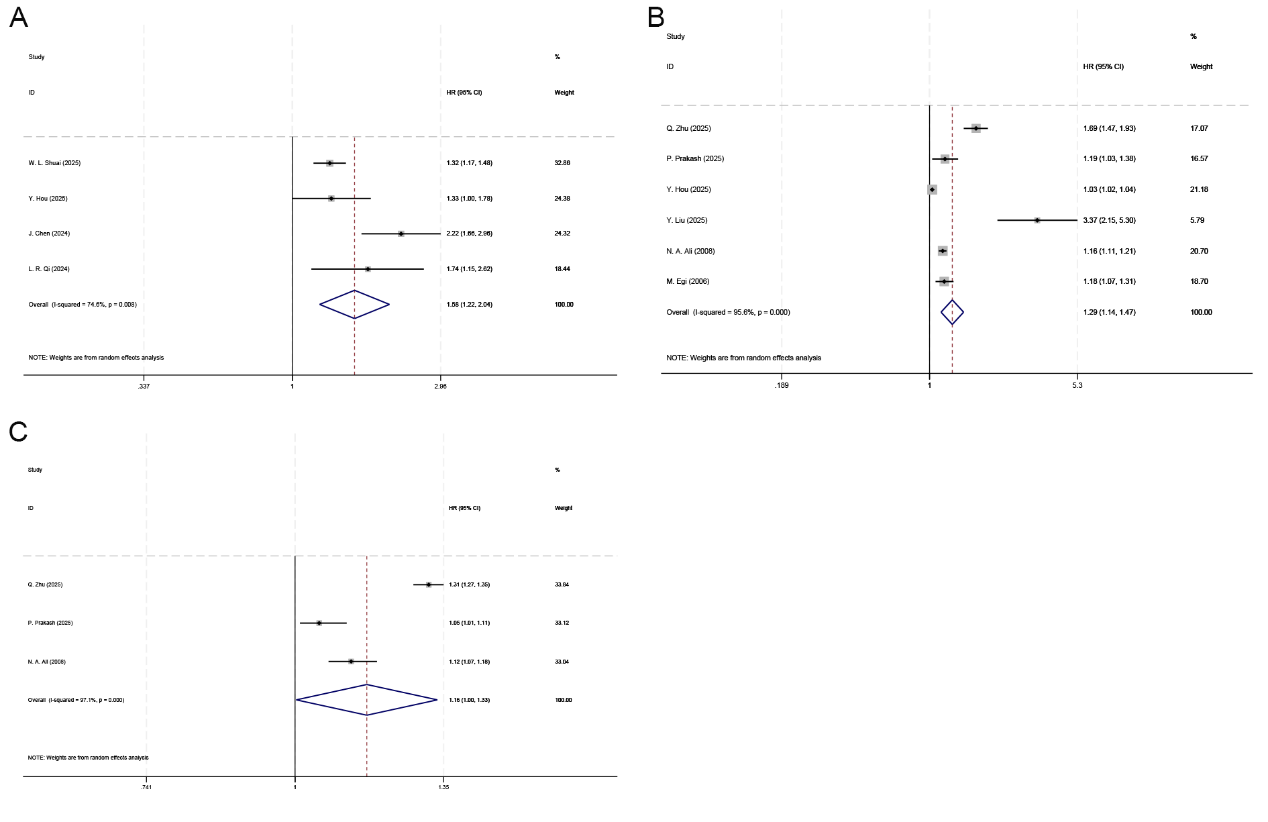


Figure 1 Forest plot of the association of GV with IHM in the critically ill population. Note: A: CVcat; B: CVcon; C: MAGE.


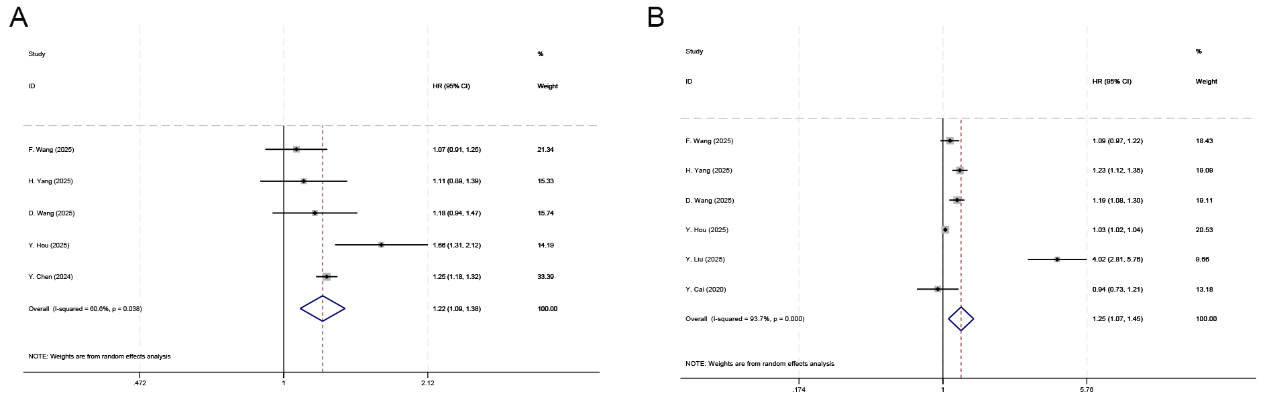


Figure 2 Forest plot of the association of GV with 90-day ACM in the critically ill population. Note: A: CVcat; B: CVcon.


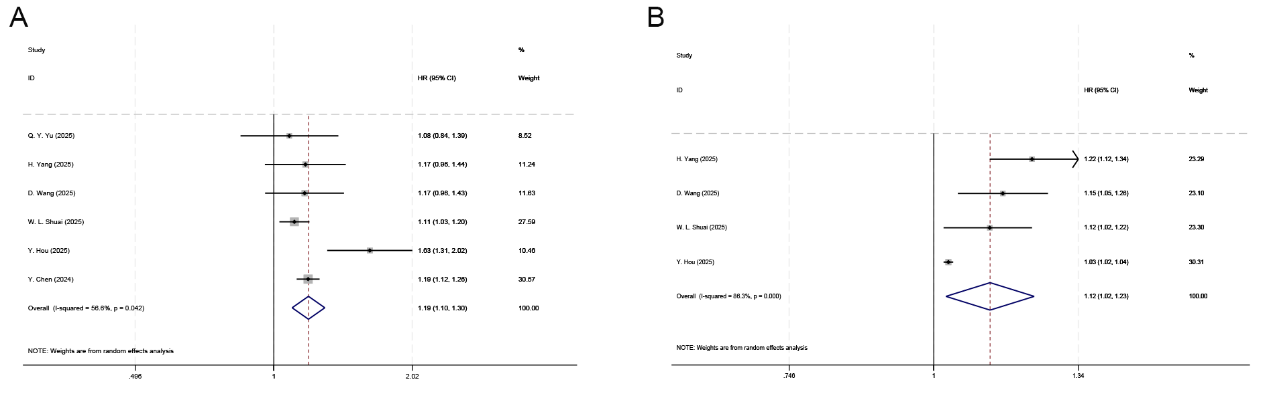


Figure 3 Forest plot of the association of GV with one-year ACM in the critically ill population. Note: A: CVcat; B: CVcon.


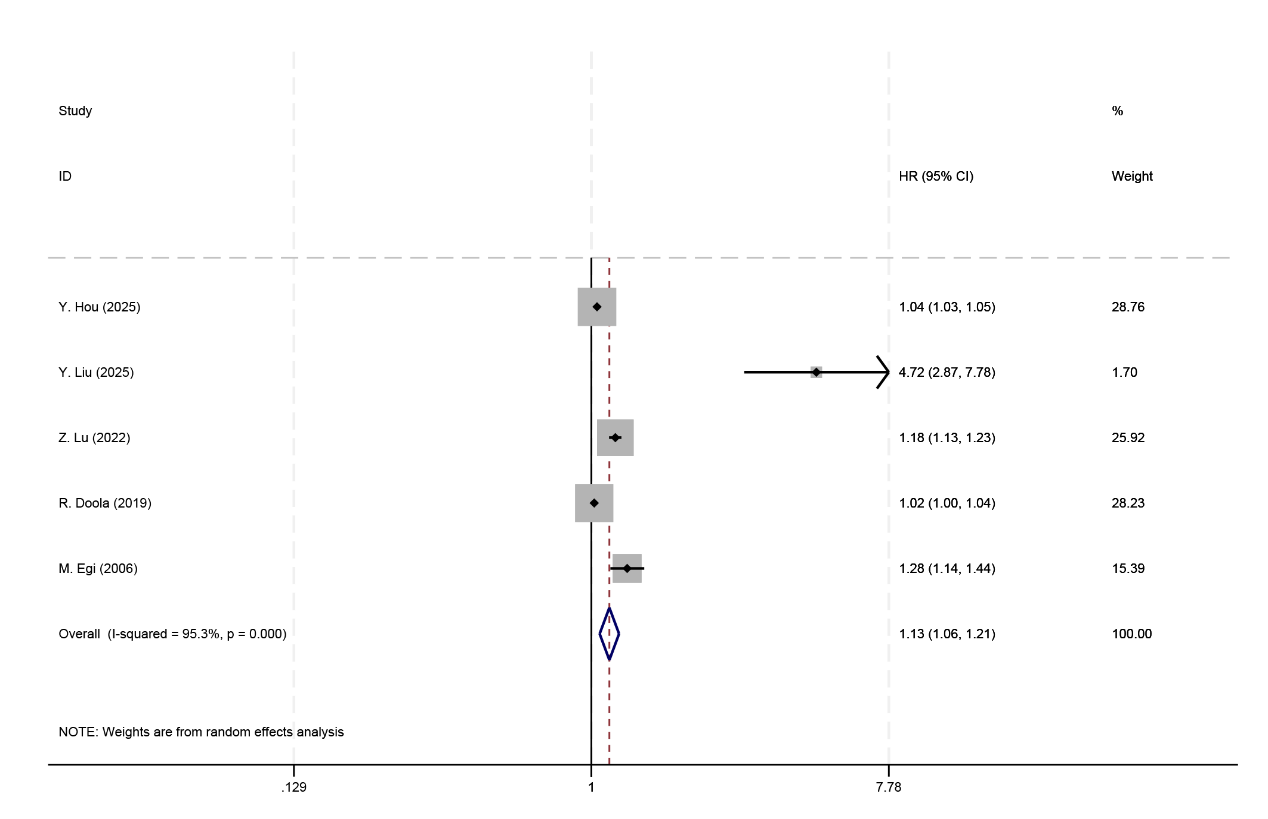


Figure 4 Forest plot of the association of GV with ICU mortality in the critically ill population.


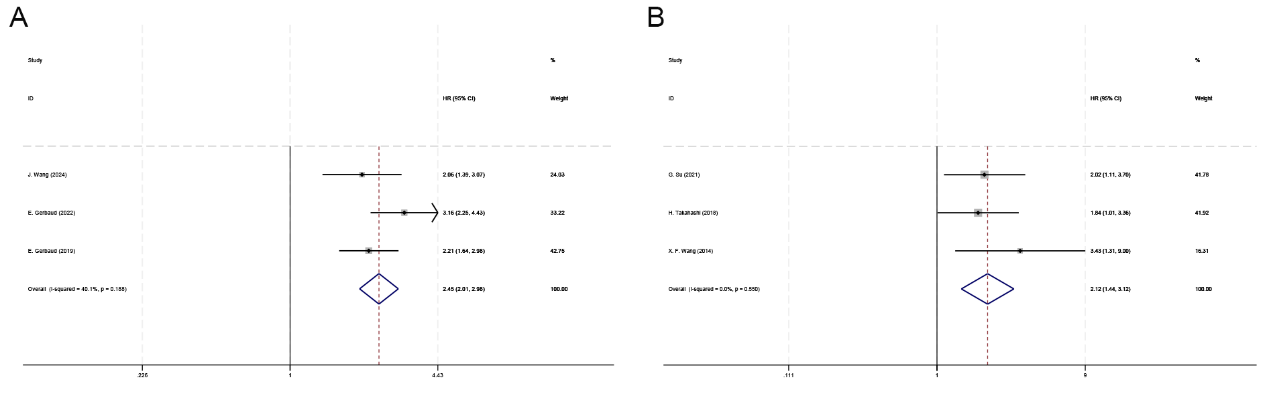


Figure 5 Forest plot of the association of GV with MACEs in the critically ill population. Note: A: SD; B: MAGE


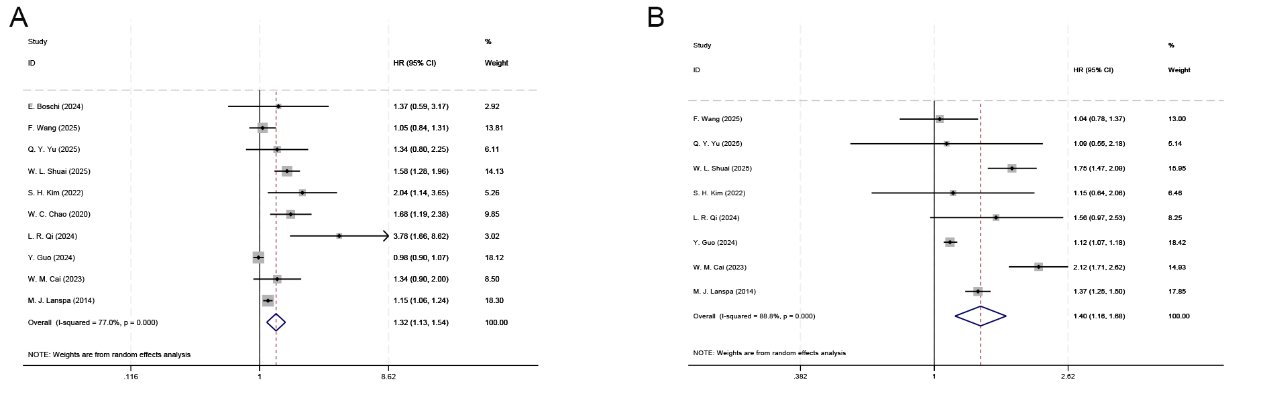


Figure 6 Forest plot of subgroup analysis based on diabetes status. Note: A: DM; B: NDM.


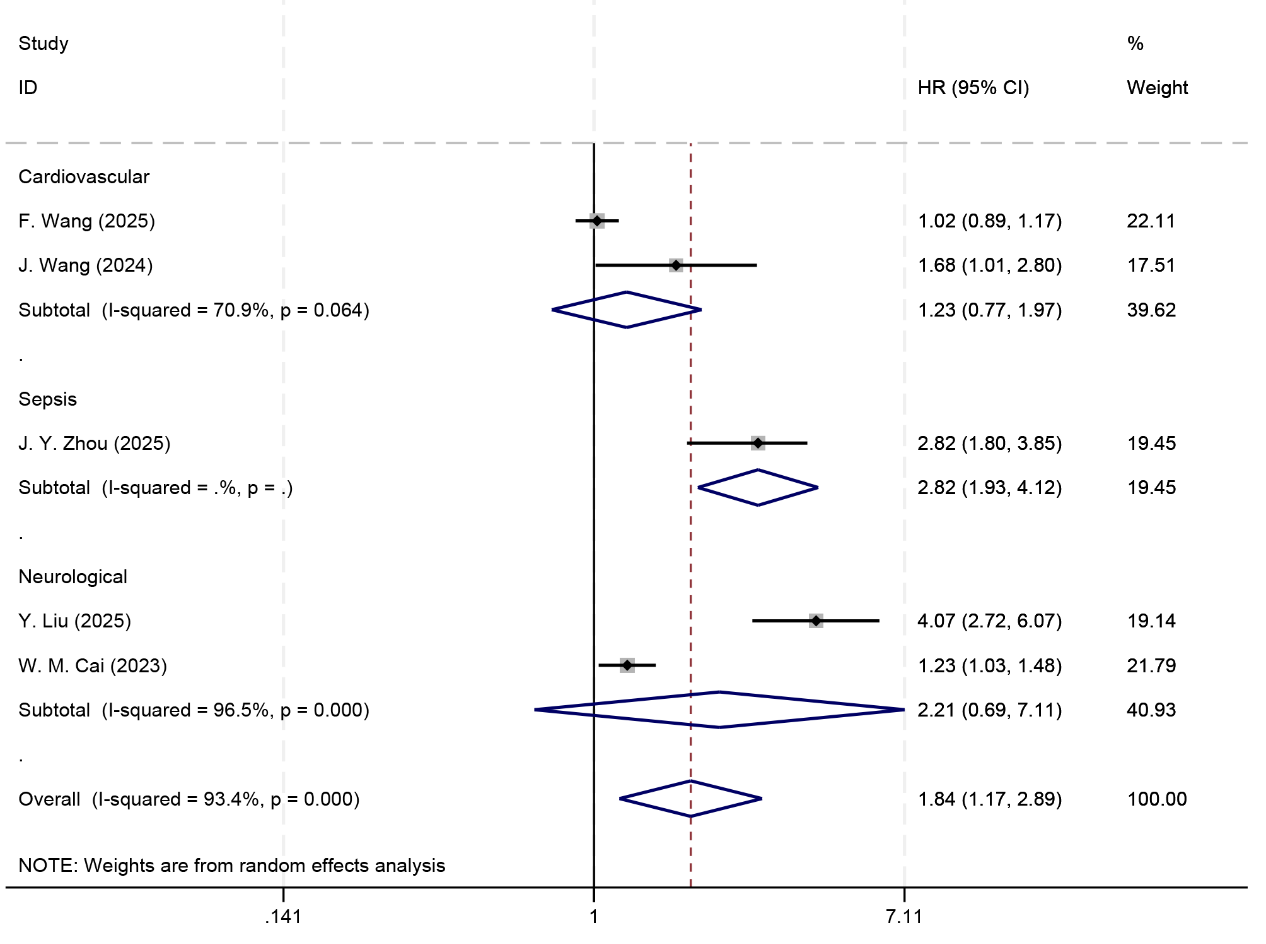


Figure 7 Forest plot of the subgroup analysis for 30-day ACM stratified by disease categories (Sepsis, Cardiovascular, and Neurological)

Sensitivity analysis results


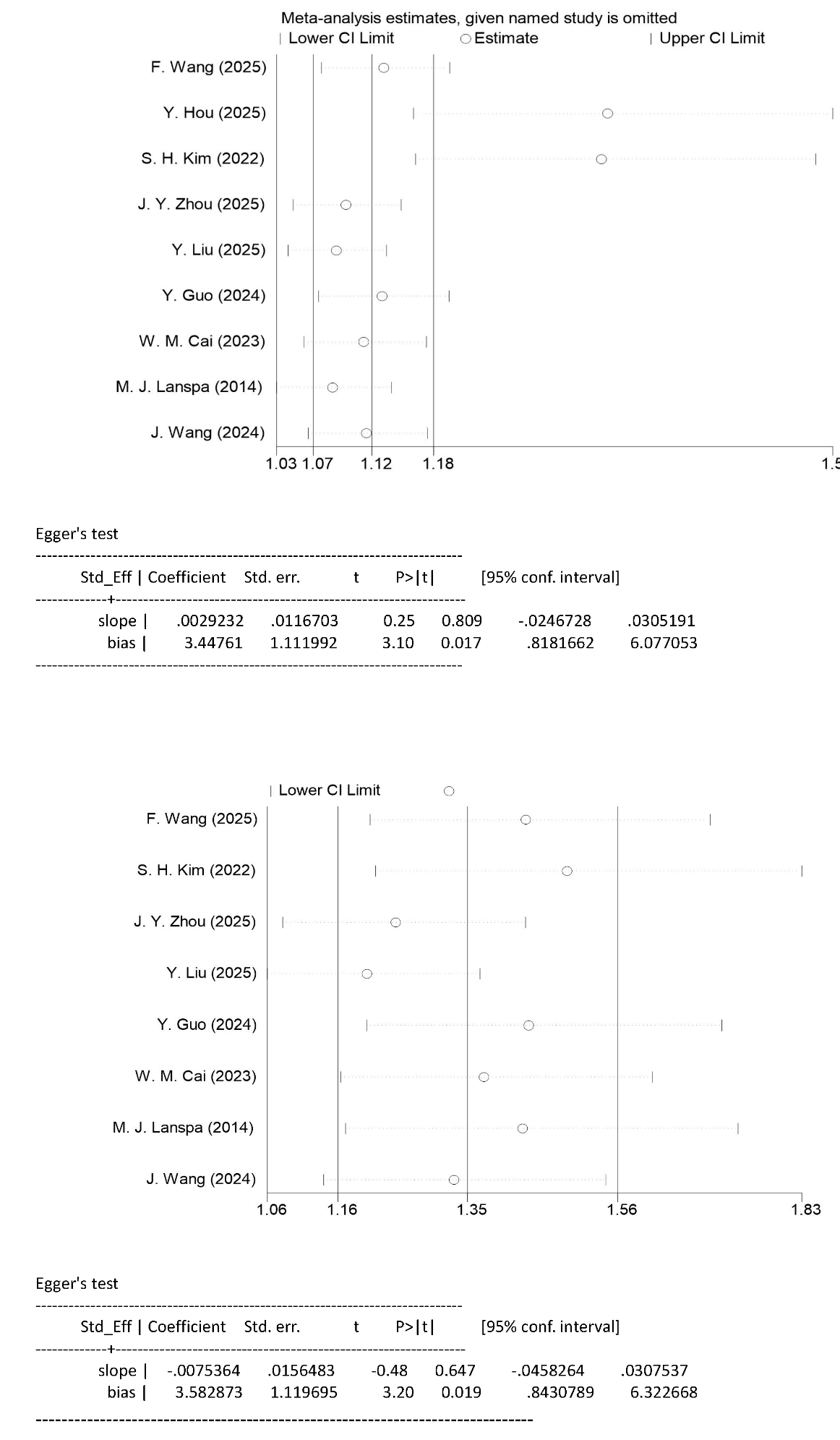


A: 30-day ACM (CVcon)


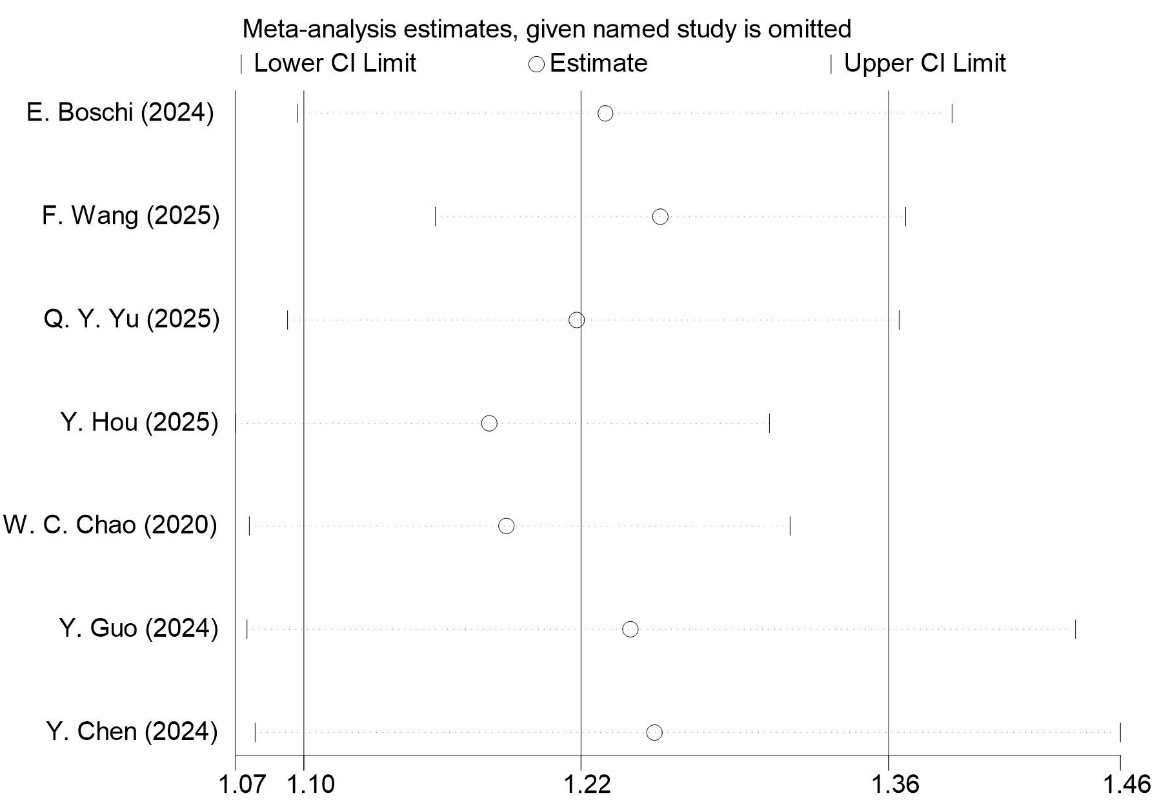


B: 30-day ACM (CVcat)


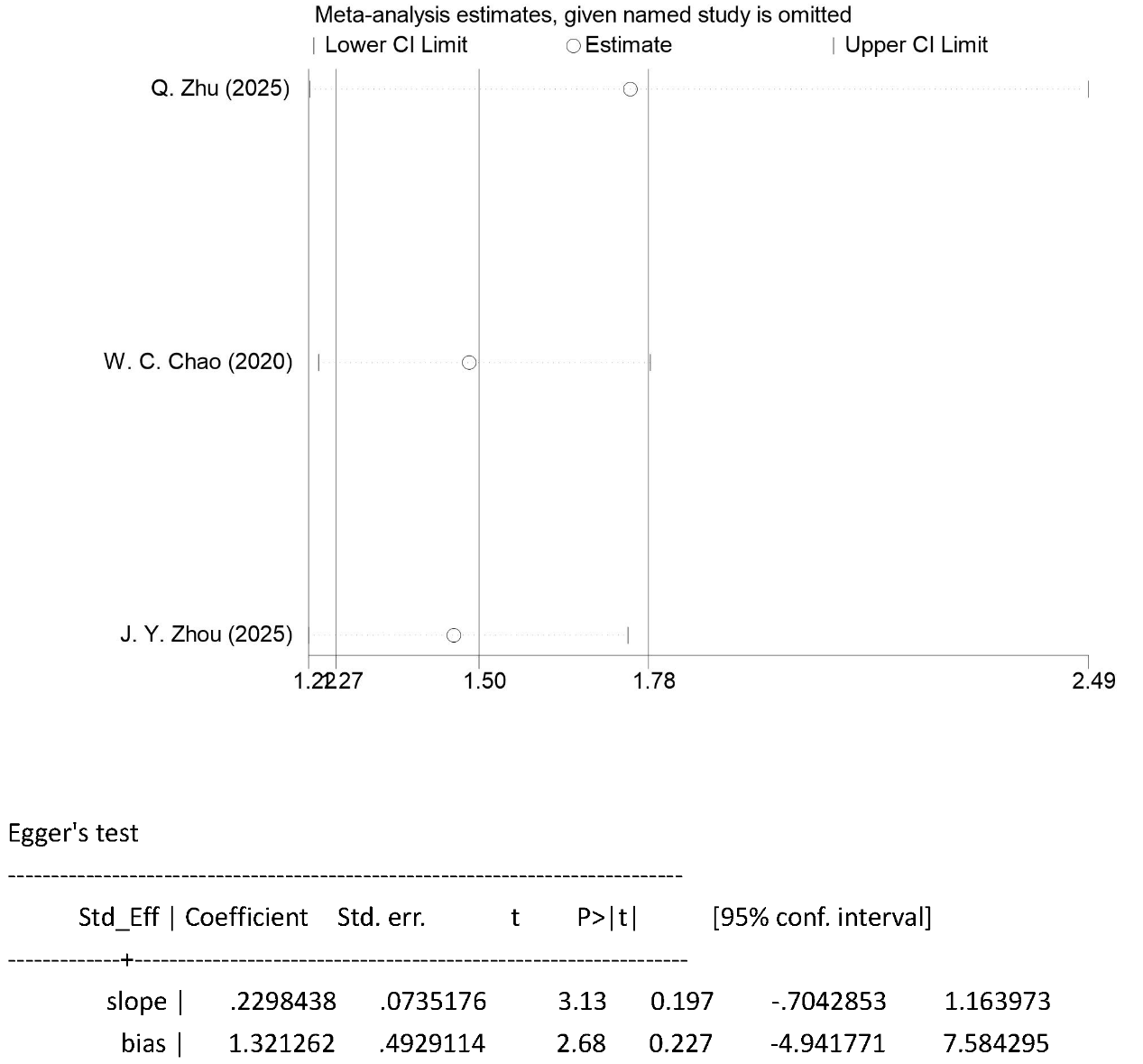


C: 30-day ACM (MAGE)


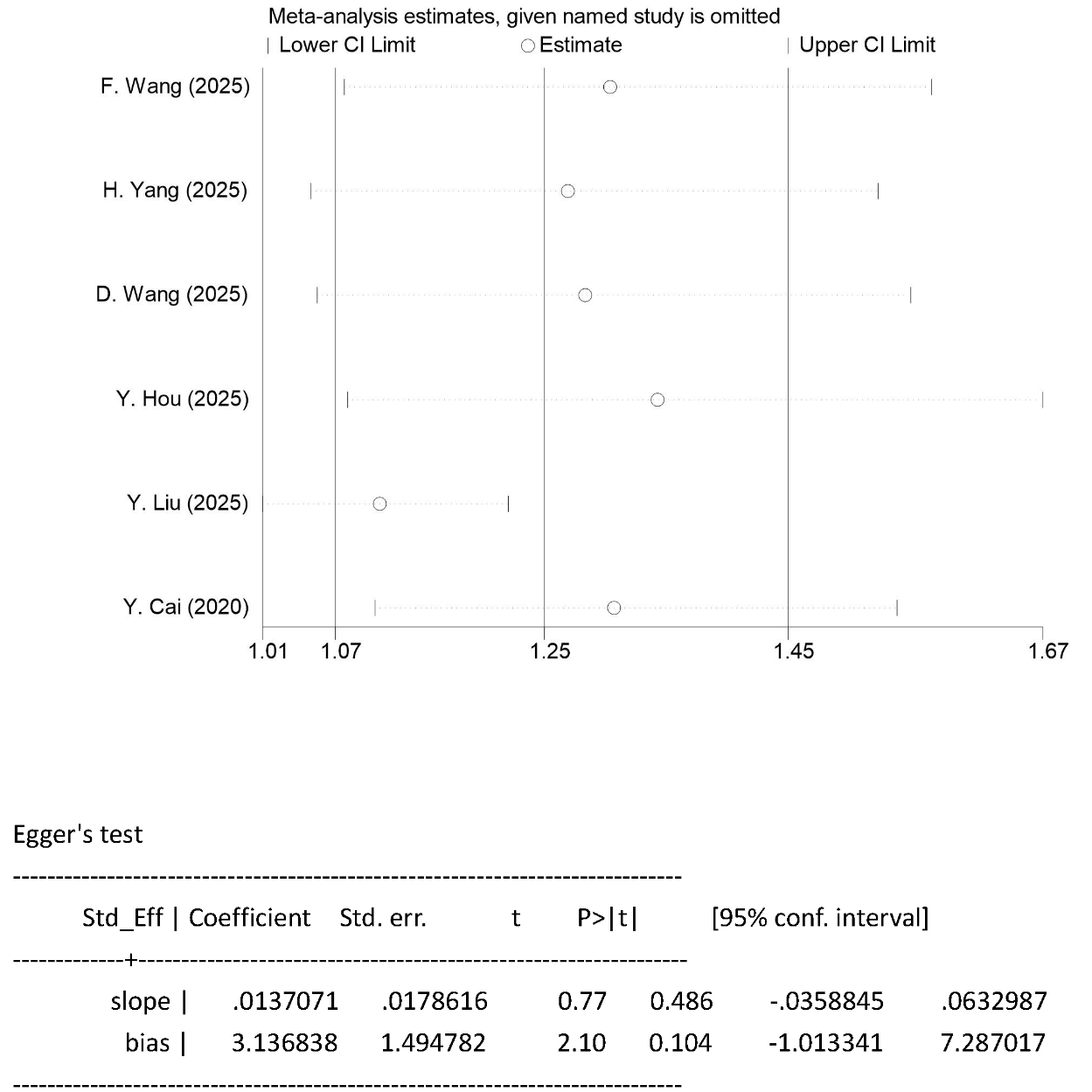


D: 90-day ACM (CVcon)


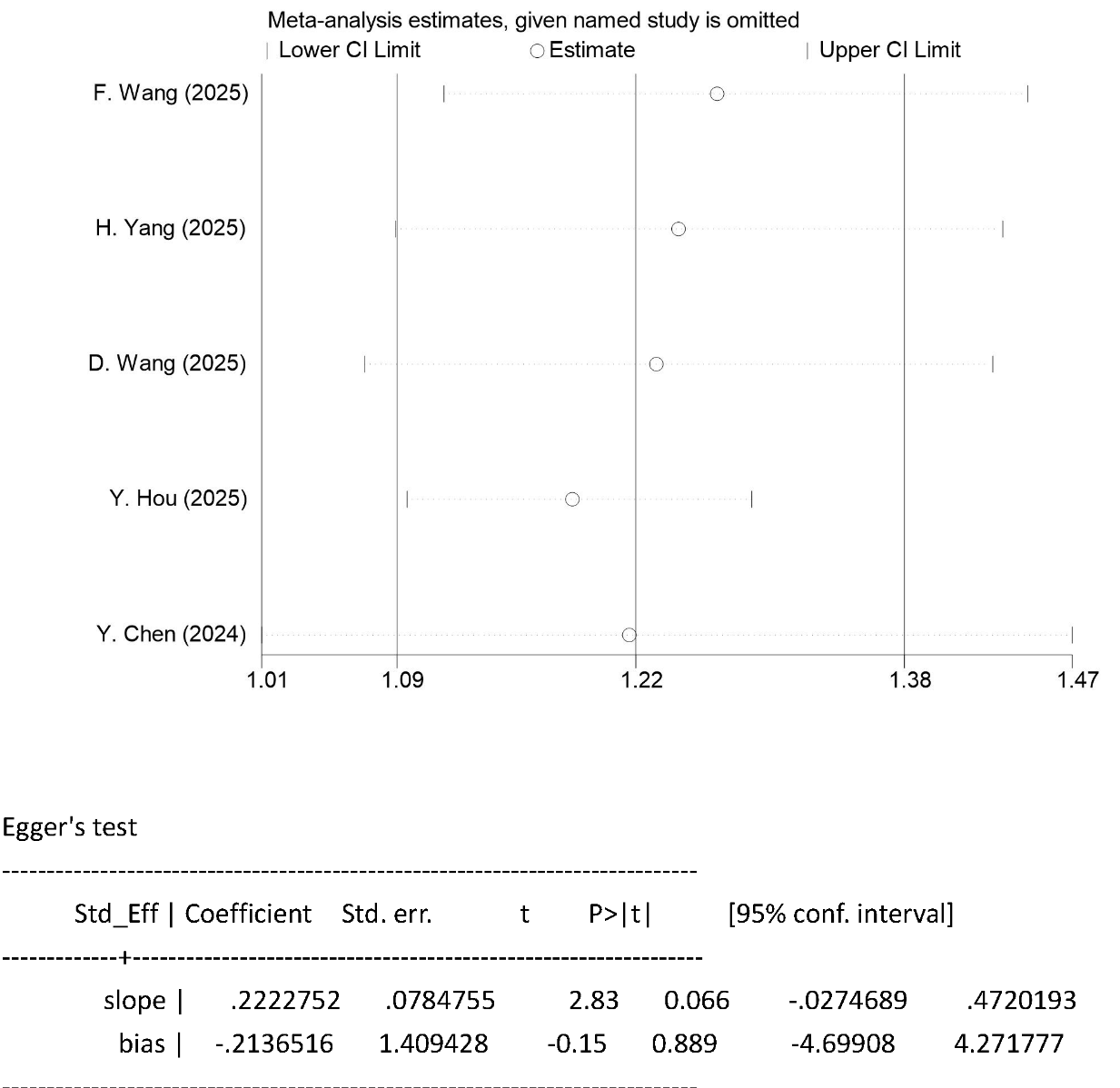


E: 90-day ACM (CVcat)


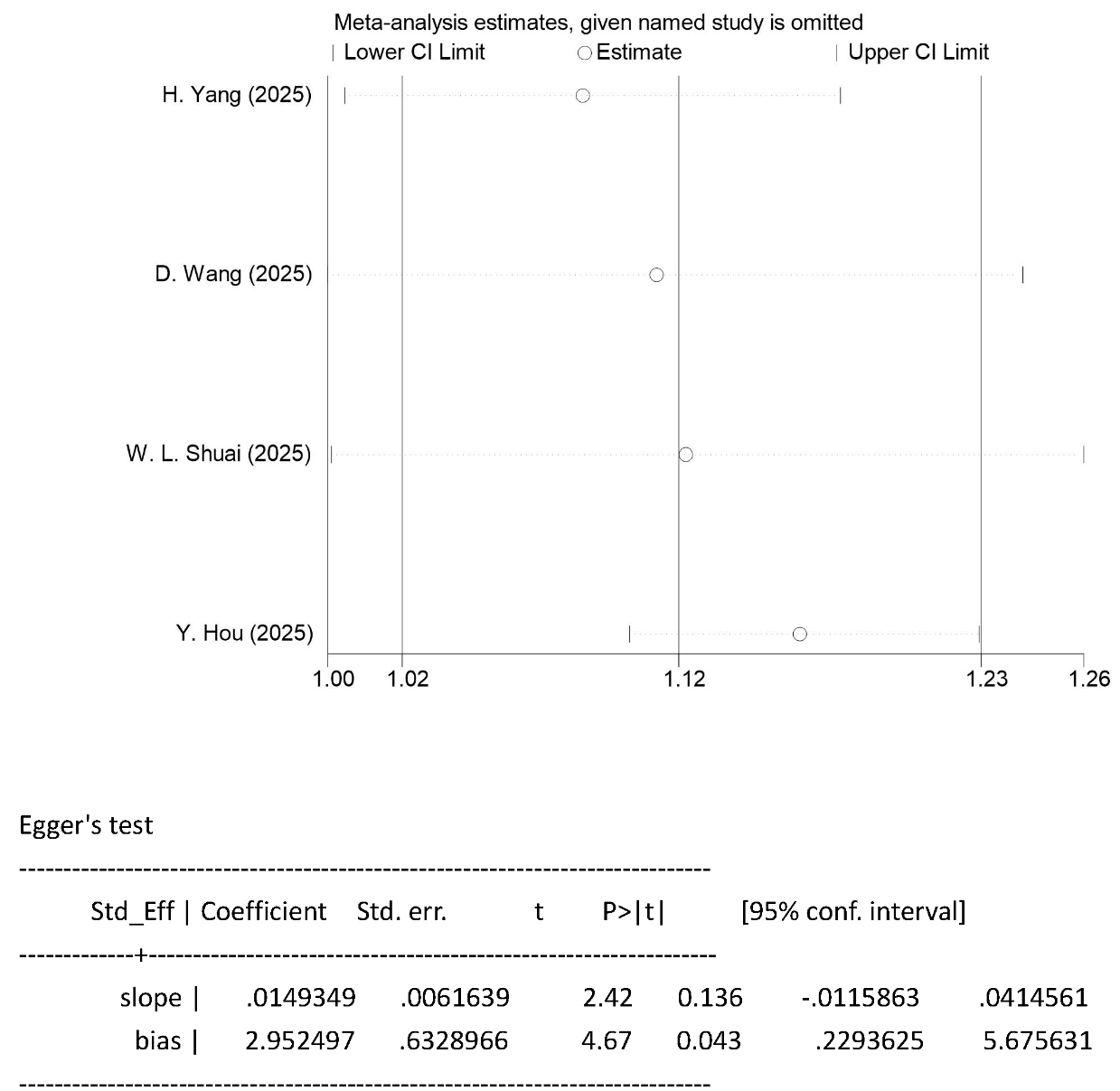


F: one-year ACM (CVcon)


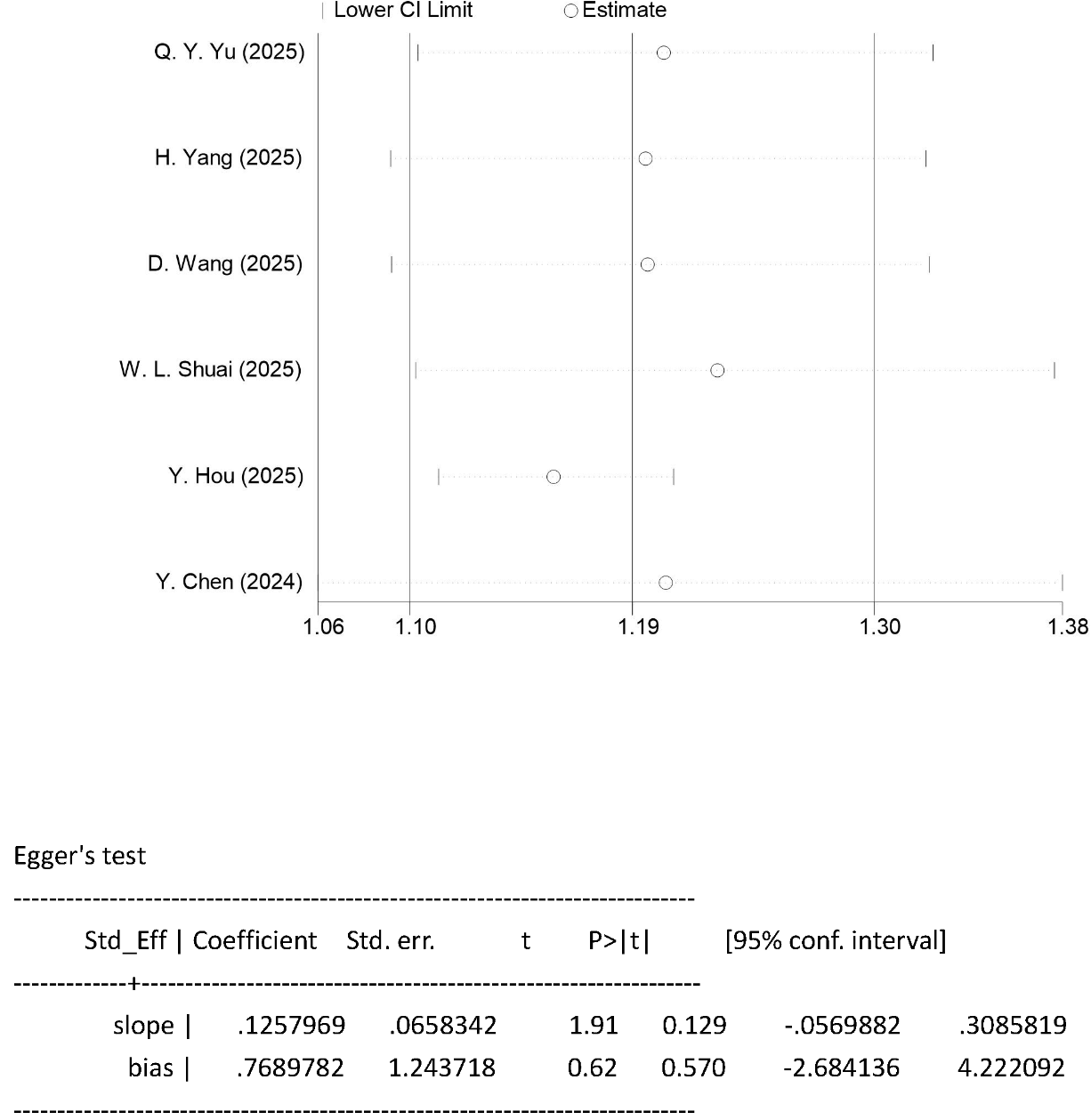


G: one-year ACM (CVcat)


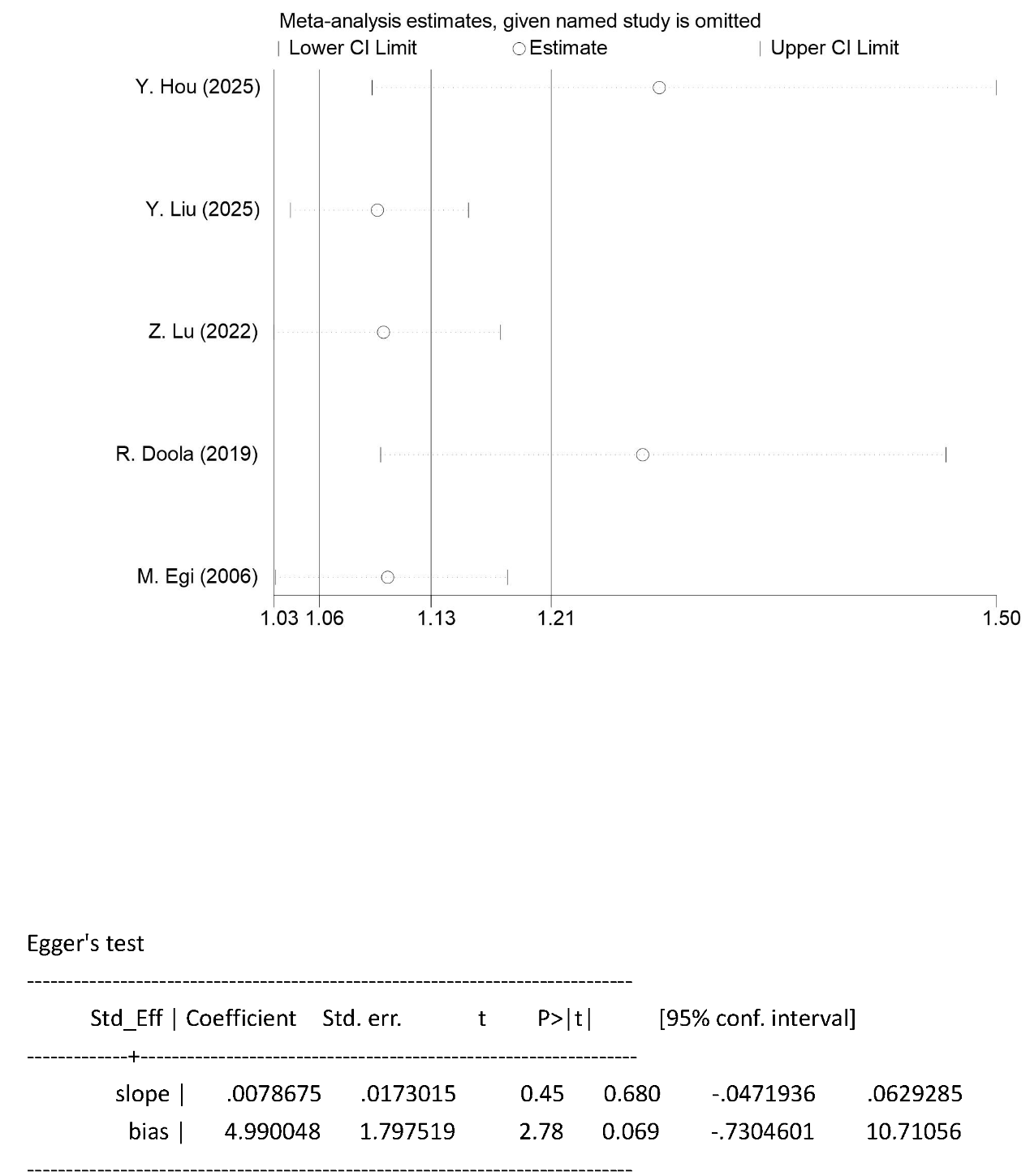


H: ICU mortality (CVcon)


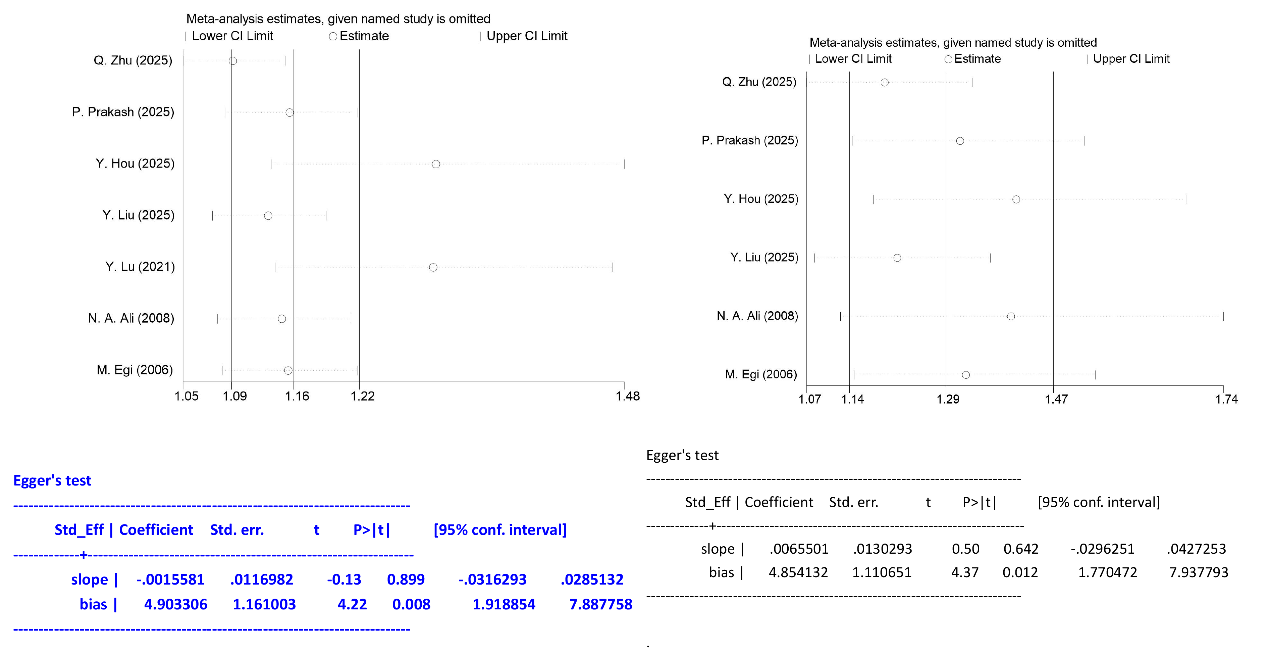


I: IHM (CVcon)


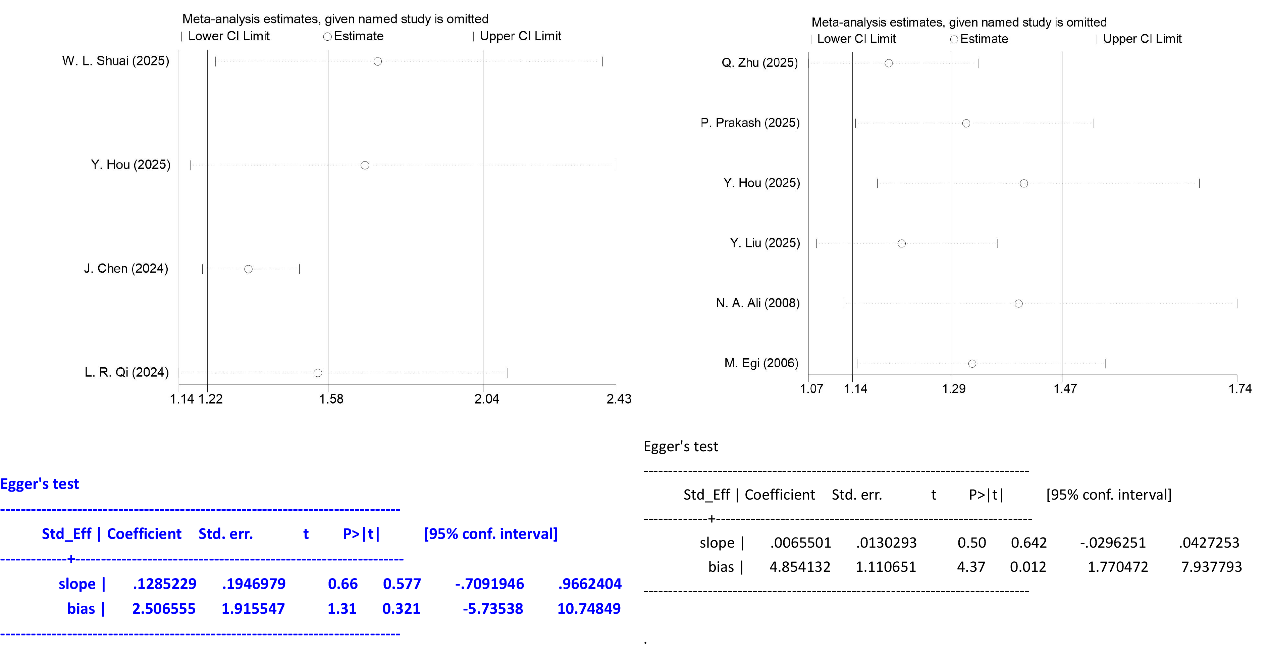


J: IHM (CVcat)


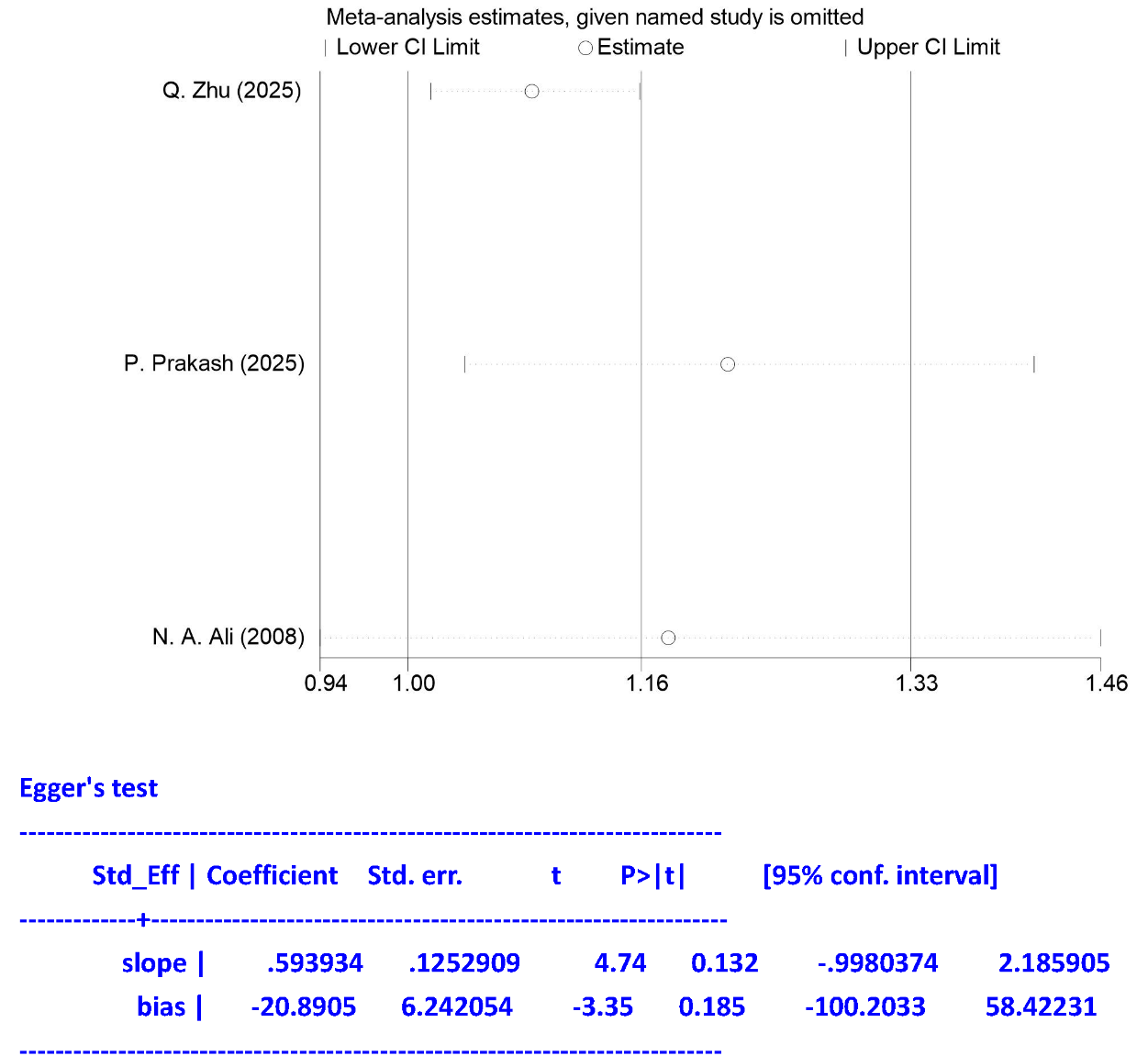


K: IHM (MAGE)


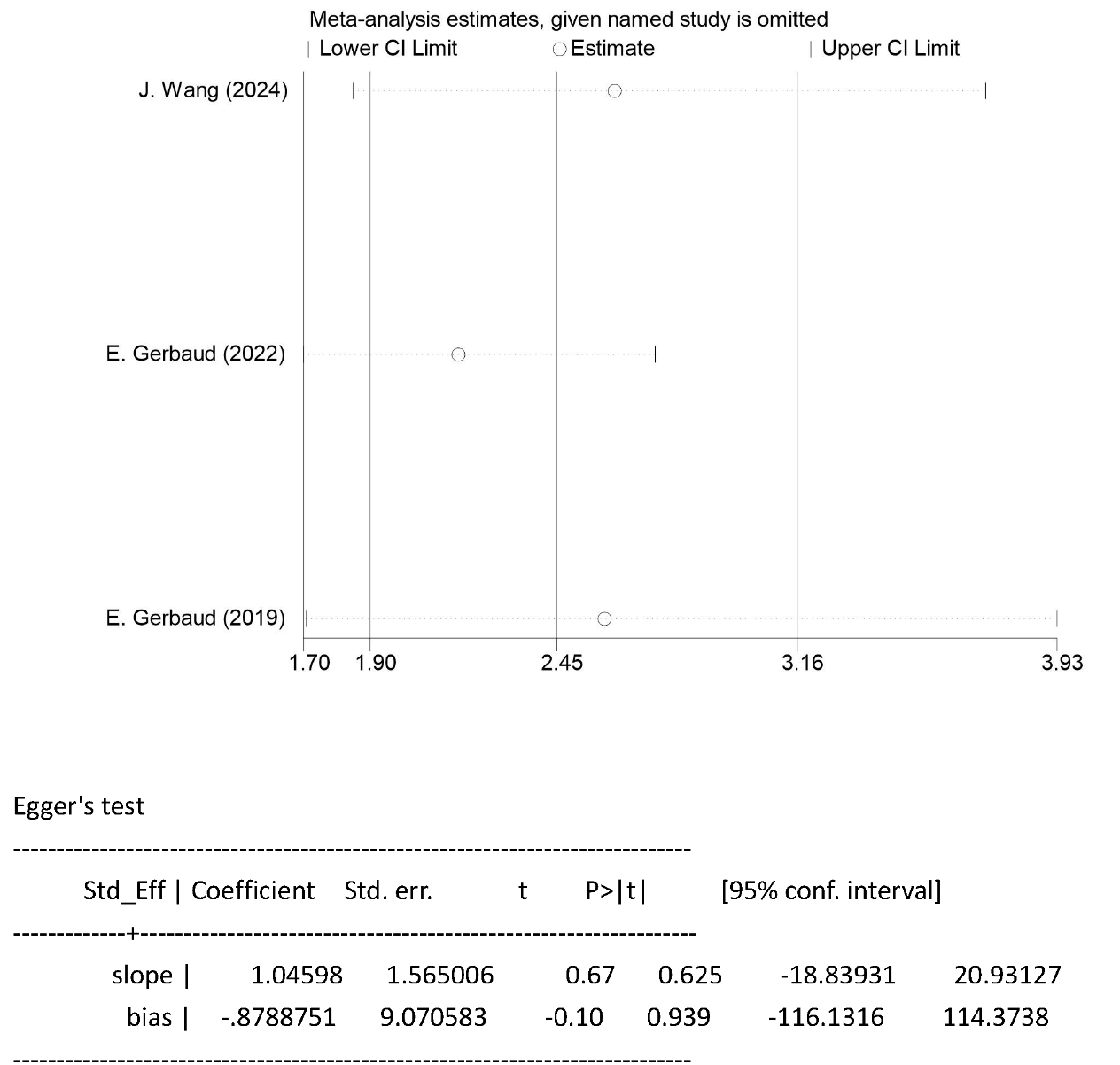


L: MACEs (SD)


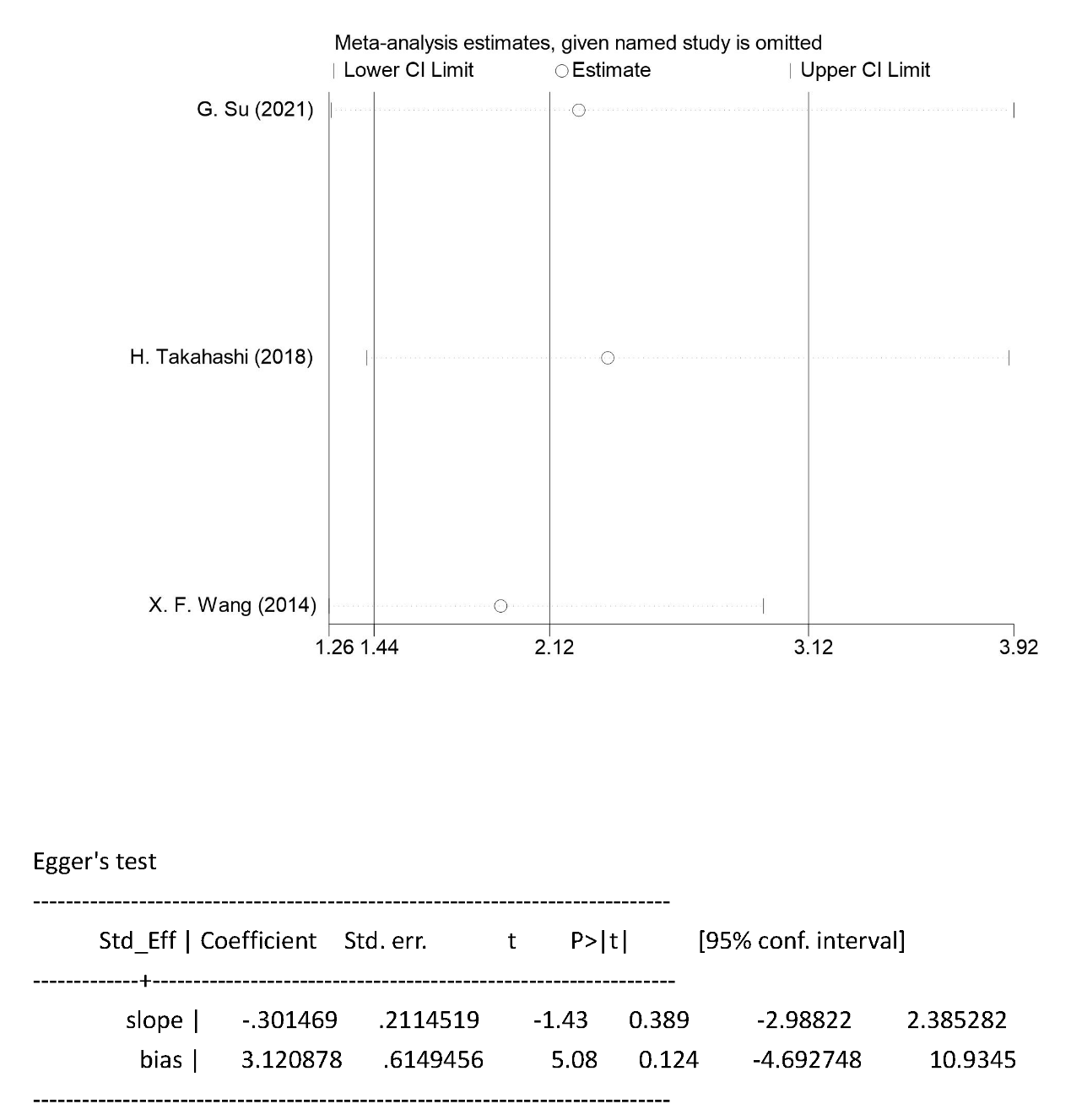


M: MACEs (MAGE)


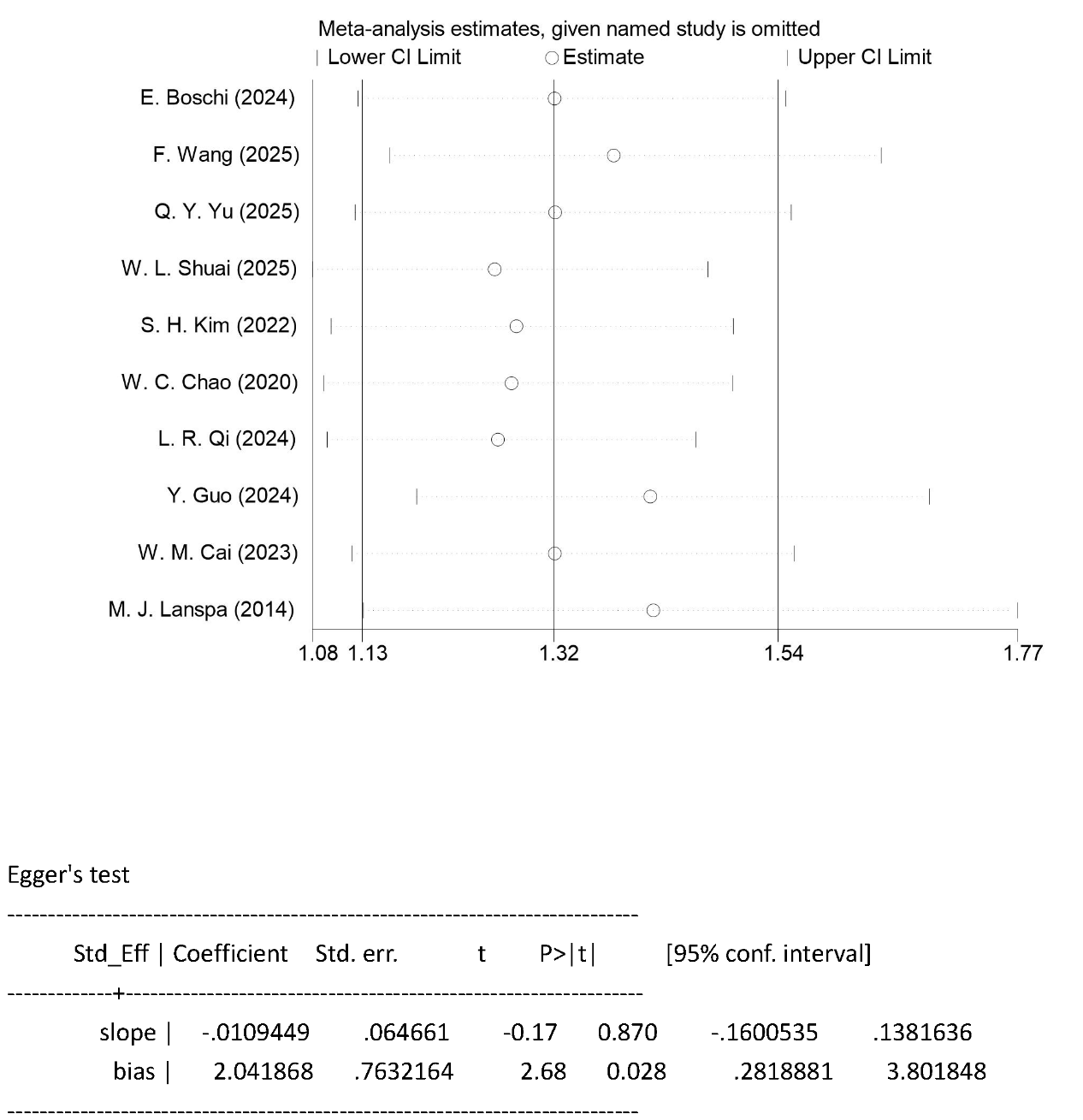


N: DM (CV)


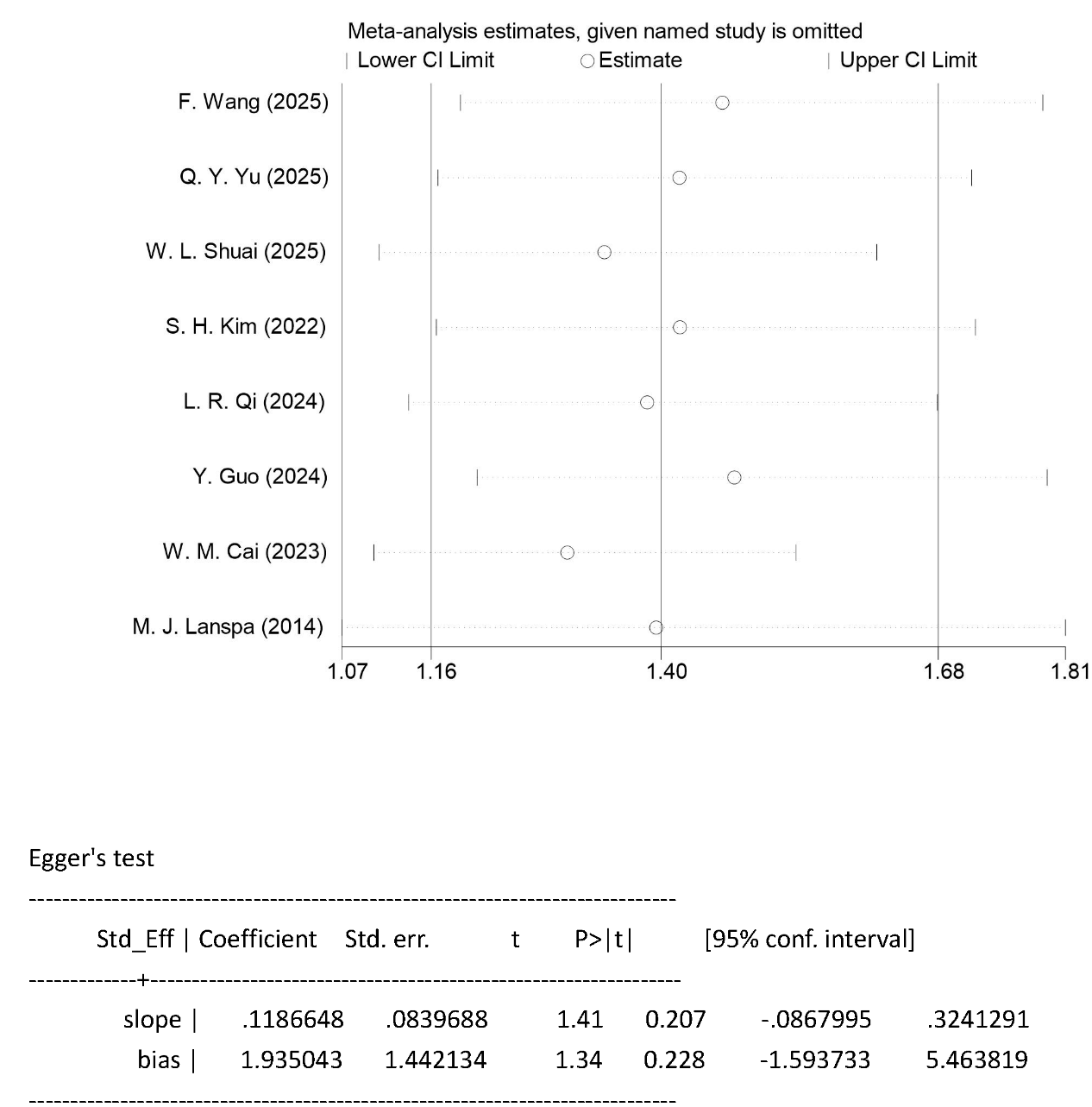


O: NDM (CV)
